# Mutations in thyroid hormone receptor α1 cause premature neurogenesis and progenitor cell depletion in human cortical development

**DOI:** 10.1073/pnas.1908762116

**Published:** 2019-10-18

**Authors:** Teresa G. Krieger, Carla M. Moran, Alberto Frangini, W. Edward Visser, Erik Schoenmakers, Francesco Muntoni, Chris A. Clark, David Gadian, Wui K. Chong, Adam Kuczynski, Mehul Dattani, Greta Lyons, Alexandra Efthymiadou, Faraneh Vargha-Khadem, Benjamin D. Simons, Krishna Chatterjee, Frederick J. Livesey

**Affiliations:** ^a^Gurdon Institute, University of Cambridge, Cambridge CB2 1QN, United Kingdom;; ^b^Cavendish Laboratory, University of Cambridge, Cambridge CB3 0HE, United Kingdom;; ^c^Wellcome Trust-MRC Institute of Metabolic Science, University of Cambridge, Cambridge CB2 0QQ, United Kingdom;; ^d^Dubowitz Neuromuscular Centre and National Institute for Health Research (NIHR) Great Ormond Street (GOS) Hospital Biomedical Research Centre, London WC1N 1EH, United Kingdom;; ^e^Developmental Imaging and Biophysics Section, University College London (UCL) GOS Institute of Child Health, London WC1N 1EH, United Kingdom;; ^f^Department of Radiology, Great Ormond Street Children’s Hospital, London WC1N 3JH, United Kingdom;; ^g^Department of Neuropsychology, Great Ormond Street Children’s Hospital, London WC1N 1EH, United Kingdom;; ^h^Department of Endocrinology, Great Ormond Street Children’s Hospital and Genetics and Genomic Medicine Programme, UCL GOS Institute of Child Health, London WC1N 1EH, United Kingdom;; ^i^Department of Endocrinology, University of Ioannina, 45110 Ioannina, Greece;; ^j^Cognitive Neuroscience and Neuropsychiatry Section, UCL GOS Institute of Child Health, London WC1N 1EH, United Kingdom;; ^k^UCL Great Ormond Street Institute of Child Health, London WC1N 1EH, United Kingdom

**Keywords:** thyroid hormone, brain development, iPSCs

## Abstract

Mutations in the thyroid hormone receptor α 1 gene (*THRA*) have recently been identified as a cause of intellectual deficit in humans. Patients present with structural abnormalities including microencephaly, reduced cerebellar volume and decreased axonal density. Here, we show that directed differentiation of *THRA* mutant patient-derived induced pluripotent stem cells to forebrain neural progenitors is markedly reduced, but mutant progenitor cells can generate deep and upper cortical layer neurons and form functional neuronal networks. Quantitative lineage tracing shows that *THRA* mutation-containing progenitor cells exit the cell cycle prematurely, resulting in reduced clonal output. Using a micropatterned chip assay, we find that spatial self-organization of mutation-containing progenitor cells in vitro is impaired, consistent with down-regulated expression of cell–cell adhesion genes. These results reveal that thyroid hormone receptor α1 is required for normal neural progenitor cell proliferation in human cerebral cortical development. They also exemplify quantitative approaches for studying neurodevelopmental disorders using patient-derived cells in vitro.

## Significance

Thyroid hormone deficiencies are the most common preventable causes of intellectual disability. We report that mutations in the thyroid hormone receptor α1 gene (*THRA*) that result in intellectual disability also reduce brain size. Using human *THRA* mutation stem cell models, we studied the impact of *THRA* mutations on human brain development by combining quantitative lineage analysis, gene expression analyses, and novel assays of neuroepithelium formation. We found that *THRA* regulates the balance between progenitor self-renewal and neurogenesis, and thus overall brain size. Importantly, these in vitro results are consistent with in vivo evidence from magnetic resonance imaging of people with these mutations, advancing our understanding of thyroid hormone action in human brain development.

The human cerebral cortex mediates higher cognitive and sensorimotor functions, with thyroid hormone (TH) deficiency during pregnancy or the neonatal period recognized as the most common preventable cause of intellectual disability worldwide (1). Defects in progenitor cell proliferation, synaptogenesis, and dendritic arborization, neuronal migration, and cell survival have been observed in the cerebral cortex of the progeny of hypothyroid rodents (2–4). Aberrant behavior and cortical cytoarchitecture are observed even following transient TH deficiency during the first half of gestation, emphasizing the critical role of THs in early brain development (5). However, in humans, the actions of THs on cells of the central nervous system (CNS) remain poorly defined (6). In the absence of appropriate in vitro models, it has been difficult to study TH action in specific cells or tissues separate from its global effects, which are likely mediated by a range of tissues and cell types (7).

During cerebral cortex development, THs (thyroxine, T4; triiodothyronine, T3) act via a nuclear receptor (TRα1) encoded by the *THRA* gene, to regulate transcription of target genes in a ligand-dependent manner (8–10). Unliganded TH receptors (TRs) recruit a corepressor complex to inhibit target gene transcription (11); hormone (T3) occupancy promotes dissociation of the corepressor complex together with coactivator recruitment and transcriptional activation (11, 12).

We reported the first human *THRA* mutation in 2012 (13), after which approximately 29 other patients have been identified with shared phenotypic features defining the disorder resistance to thyroid hormone α (RTHα) (14–18). All the patients carry heterozygous missense or truncating mutations in the ligand- binding domain of TRα1 that disrupt its ability to bind T3, impairing corepressor dissociation and coactivator recruitment (13, 16). When coexpressed, mutant TRα1 inhibits the function of its wild-type (WT) counterpart in a dominant-negative manner (13). In addition to growth retardation and skeletal dysplasia, patients with RTHα exhibit mild-to-moderate intellectual disability, notably affecting nonverbal IQ and sensorimotor processing, and 1 adult woman has experienced epileptic seizures that began in infancy (16). These findings suggest a crucial role for TRα1 in human cortical neurogenesis, consistent with previous studies reporting a range of CNS abnormalities in mice mutant for TRα1 (19). However, the cellular mechanisms underlying aberrant neural development in patients with RTHα remain unknown.

Here we have delineated the neurologic and neurocognitive phenotypes and undertaken structural (magnetic resonance imaging [MRI], tractography) neuroimaging and proton magnetic resonance spectroscopy (MRS) in the first 4 RTHα patients reported, harboring frameshift/premature stop *THRA* mutations that are representative of the type of receptor defect found in 50% of the worldwide RTHα cohort (20). We directed differentiation of *THRA* mutant patient-derived induced pluripotent stem cells (iPSCs) to a cortical excitatory neuronal fate, using an established in vitro system that recapitulates development from early neuroepithelium to functional neuronal circuits (21, 22). Based on quantitative analysis of lineage tracing data, we found that *THRA* mutation-containing cortical progenitor cells are biased toward early differentiation, leading to premature neurogenesis and depletion of the progenitor cell pool. They also exhibit impaired self-organization into cortical rosette-like structures in vitro. Defects in neural progenitor proliferation, cell polarity, and apical adhesion may thus contribute to the structural abnormalities and to the sensorimotor and neurocognitive phenotypes seen in patients with RTHα.

## Results

### Neurologic, Neurocognitive, and Neuroimaging Abnormalities in Patients with *THRA* Mutation.

**Fig. 1.**
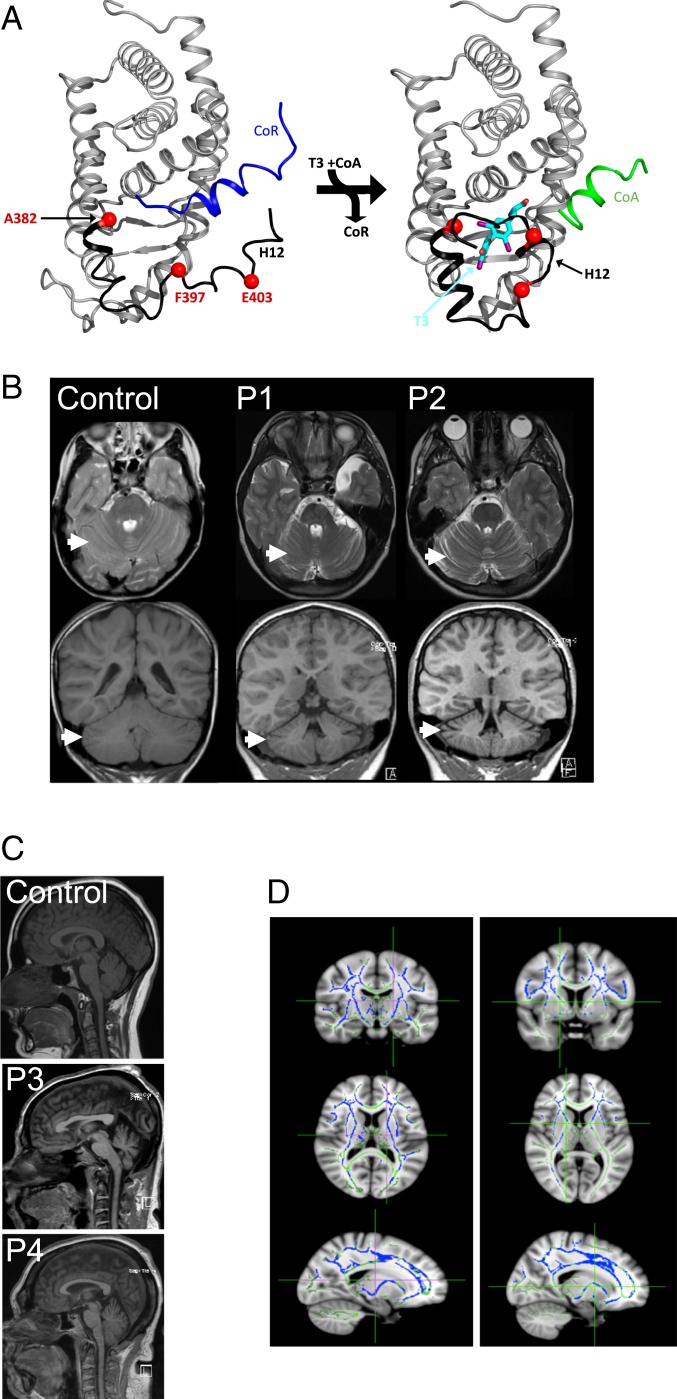
*THRA* mutations are associated with structural abnormalities in the brain. (*A*) Structural modeling of the ligand-binding domain of TRα1 showing the position of mutations (red). The mutations in patients P4 (A382PfsX7), P2 and P3 (F397fs406X), and P1 (E403X) disrupt or truncate the carboxyterminal alpha helix (H12; black) of the receptor, exposing a hydrophobic cleft that facilitates corepressor (CoR; blue) binding by unliganded receptor (*Left*) and removing or changing amino acids required for T3 (cyan) binding and coactivator (green) recruitment (*Right*). (*B*) MRI scans of patients P1 and P2 and a control subject (female, age 10 y 8 mo) with T2-weighted axial images (*Top*) and T1-weighted coronal images (*Bottom*), showing increased CSF space (arrows) around the cerebellum and between folia, denoting reduced cerebellar size. (*C*) Sagittal images from MRI brain scans of adult cases P3 and P4 and a control subject (female, age 52 y) showing microencephaly. (*D*) Tract-based spatial statistics analysis of DTI data in patients P1 and P2. Tracts highlighted in blue signify greater mean diffusivity (MD) of water than in controls (*n* = 20 age- and sex-matched subjects), and tracts highlighted in green denote not significantly different MD compared with controls.

We assessed neurologic, neurocognitive, and neuroimaging phenotypes in the first 4 RTHα cases reported (*SI Appendix*, Table S1), all harboring mutations that disrupt the carboxyterminal alpha helix of TRα1 (Fig. 1*A*). The subjects are a 9-y-old female (E403X TRα1 mutation; referred to as P1 below), a 13-y-old female and her 49-y-old father (F397fs406X TRα1 mutation; P2 and P3), and a 47-y-old female (A382PfsX7 TRα1 mutation; P4). All 4 patients exhibited delayed developmental milestones (*SI Appendix*, Table S2) and neurologic abnormalities, including slow initiation of movement, ataxic gait, dysarthria, and fine and gross motor incoordination (*SI Appendix*, Table S3).

Neuropsychological examinations showed significantly reduced nonverbal IQ in all cases, with scores ranging from 2 (P1, P2, and P3) to 3.4 (P4) SDs below the population mean (*X* = 100; SD, 1.5). Furthermore, all patients showed severe impairments in motor coordination, visual motor integration, and finger dexterity of both the dominant and nondominant hands. P4 showed intellectual disability affecting verbal as well as nonverbal abilities, whereas verbal abilities were relatively preserved in P1, P2, and P3. Performance on a test of visual perception was within the low average to average range in P1-P3 (*SI Appendix*, Table S4).

Magnetic resonance imaging (MRI) revealed reduced cerebellar volumes in all 4 patients (Fig. 1*B* and *SI Appendix*, Fig. S1), with microencephaly in the adult cases (P3 and P4), despite their increased skull volume (macrocephaly) denoted by known increased head circumference (P1, +3 SDs from the population mean; P2, +4 SD; P3, +5 SD; P4, +9 SD) (Fig. 1*C*). Diffusion tensor imaging (DTI) performed in P1 and P2 showed a global increase in the mean diffusivity of water in white matter tracts, denoting a reduction in their density (Fig. 1*D*). On MRS, there was a reduced ratio of *N*-acetylaspartate to the creatine plus phosphocreatine ratio in the frontal white matter and thalamus in P2, P3, and P4, suggestive of neuronal loss or dysfunction (*SI Appendix*, Table S5). These results suggest that the observed neurocognitive and sensorimotor deficits in the patients are related to structural abnormalities in several brain regions, including the cerebellum and cerebral cortex.

### Reduced Differentiation of *THRA* Mutant iPSCs to Forebrain Neuroepithelial Cells.

**Fig. 2.**
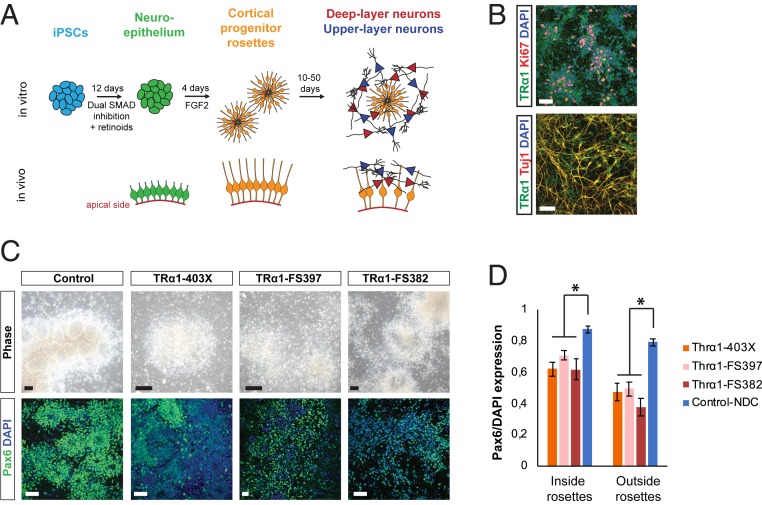
Inefficient cortical induction of *THRA* mutation-containing iPSCs. (*A*) Schematic of the in vitro cortical differentiation protocol. (*B*) TRα1 is expressed in cycling Ki67^+^ progenitors as well as postmitotic Tuj1^+^ neurons derived from control iPSCs. (Scale bars: 50 μm.) (*C*) Representative pictures showing reduced progenitor rosette formation and Pax6 expression in *THRA* mutation-containing cells compared with control cultures at day 15. At least 3 independent differentiations were performed for 1 clone from each patient. (Scale bars: 50 μm.) (*D*) Quantification of Pax6 expression within and outside of neural progenitor cell rosettes in *THRA* mutant and control cell cultures at day 15 of neural induction. Rosettes were manually delineated based on morphology in 8 or 9 images from 2 or 3 independent inductions for each cell line, and Pax6 expression was measured relative to DAPI (*Methods*). Error bars indicate SEM. *P < 0.001, 2-sided Student’s *t* test comparing a total of nine control and 24 *THRA* mutant images.

To study the cellular developmental origin of cortical abnormalities in *THRA* mutant patients, iPSC lines were derived from P1, P2, and P4 by reprogramming with nonintegrating Sendai virus (*SI Appendix*, Fig. S2*A*) (23). *THRA* mutant and control iPSCs were then differentiated to cerebral cortex neural progenitor cells using an established protocol based on dual SMAD inhibition (Fig. 2*A*) (21, 22). After 12 d of induction, a neuroepithelial sheet was formed in control cultures, and expression of PAX6 and FOXG1 mRNA confirmed cortical identity (*SI Appendix*, Fig. S2*B*). Neural progenitor cells proceeded to generate neurons as described previously (22). In control cultures, TRα1 was expressed in stem cells before cortical induction, in cycling (Ki67^+^) progenitor cells, and in postmitotic (Tuj1^+^) neurons (Fig. 2*B* and *SI Appendix*, Fig. S2*C*), confirming that the receptor is present throughout the in vitro differentiation process.

When *THRA* mutation-containing iPSCs from the 3 patients were subjected to the same differentiation protocol, cortical induction was found to be reduced in efficiency. While cortical transcription factors were also expressed in *THRA* mutant cultures (*SI Appendix*, Fig. S2*B*), expression of PAX6 was highly variable between cells (*SI Appendix*, Fig. S2*D*). At day 15, following manual fragmentation of the neuroepithelial sheet, PAX6 expression in *THRA* mutation-containing cells was decreased (Fig. 2 *C* and *D*). PAX6 expression was mostly higher in, but not confined to, areas with rosette-like morphology (Fig. 2*D*).

### Altered Expression of Neural Development and Cell–Cell Adhesion Genes during Neural Differentiation of *THRA* Mutant iPSCs.

To investigate differences in induction efficiency in an unbiased manner, we performed RNA sequencing of 3 independent neural inductions from each THRA mutation-containing iPSC line (TRα1-403X carrying the E403X mutation, TRα1-FS382 with the A382PfsX7 mutation, and TRα1-FS397 with the F397fs406X mutation) and 1 neural induction from each control line (H9, NAS6, and NDC), at day 12 of cortical induction. Out of 16,773 transcripts that were detected above negligible levels in all samples, we considered 478 transcripts whose expression differed significantly between *THRA* mutation-containing and control lines (false discovery rate <0.25, independent of the sign of the fold change).

**Fig. 3.**
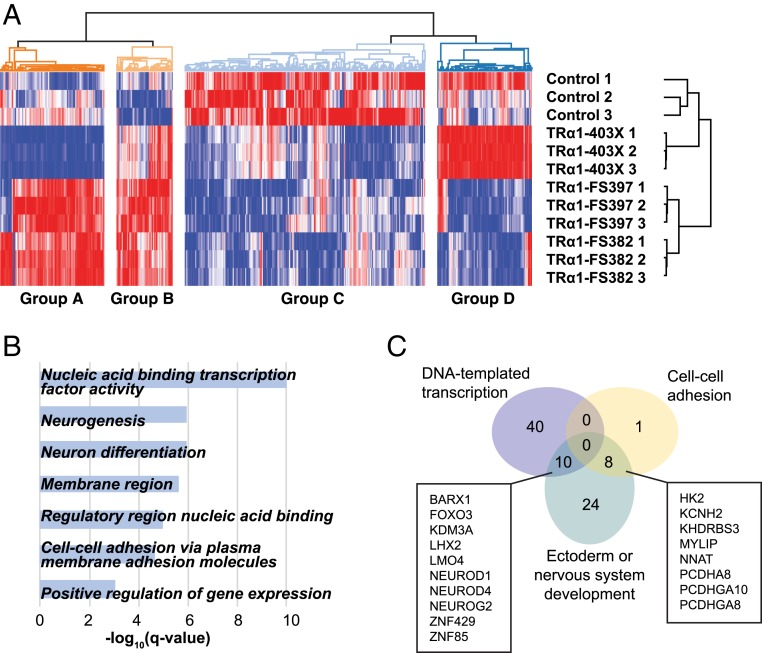
RNA-seq at day 12 of in vitro cortical induction reveals down-regulation of transcription, neurogenesis, and cell–cell adhesion in *THRA* mutation-containing cells. (*A*) 2D hierarchical clustering of log_2_(RPKM) values for 3 inductions each from control iPSCs and the 3 patient lines. Colors correspond to relative expression in each row, from lowest (blue) to highest (red). Inductions cluster by cell line of origin (vertical axis), while genes separate into 4 differentially expressed groups (horizontal axis). (*B*) FDR *q* values of GO terms enriched among group C genes. (*C*) GO analysis reveals that genes implicated in nervous system development that are down-regulated in *THRA* mutation-containing cells partially overlap with cell–cell adhesion genes (8/42; 19%) and DNA-templated transcription (10/42; 24%).

Hierarchical clustering analysis revealed that the expression profiles of replicate inductions from each *THRA* mutation-containing line clustered together, suggesting that the cortical induction process in vitro preserves mutation-specific differences in transcription (Fig. 3*A*). The differentially expressed genes clustered into 4 distinct groups, with the largest subset representing genes whose expression is down-regulated in *THRA* mutation-containing vs. control cells, consistent with the known repressive function of TRα1 (209 genes in group C). Gene Ontology (GO) analysis showed that many of these down-regulated genes relate to DNA-templated transcription, ectoderm and nervous system development, or cell–cell adhesion via membrane adhesion molecules (Fig. 3*B*), with substantial overlap (Fig. 3*C*). Genes relating to both transcription and neural development include bHLH-transcription factors such as *NEUROD1*, *NEUROD4* (*MATH3*), and *NEUROG2*, which are involved in normal cerebral cortex development (24), as well as *LHX2*, which is known to regulate the specification of cortical regional fates (25). The genes involved in both neural development and cell–cell adhesion are 6 protocadherins (part of the cadherin family), the autism susceptibility gene *NRXN1* (neurexin), and *NPTN* (neuroplastin), which has been associated with cortical thickness and intellectual ability in humans (26, 27).

Thus, our RNA-seq data show that *THRA* mutations are associated with the down-regulation of genes involved in neural development. In addition, the data suggest compromised cell–cell adhesion in *THRA* mutation-containing cells, possibly contributing to impaired cortical growth. Notably, within up-regulated (group B) genes, enrichment for nonneural differentiation fates was not detected, suggesting that the reduced efficiency of neural induction seen in *THRA* mutation-containing cells is not due to differentiation down nonneural paths.

### CD271^−^CD44^−^ Cortical Progenitor Cells Generate Functional Neurons In Vitro.

**Fig. 4.**
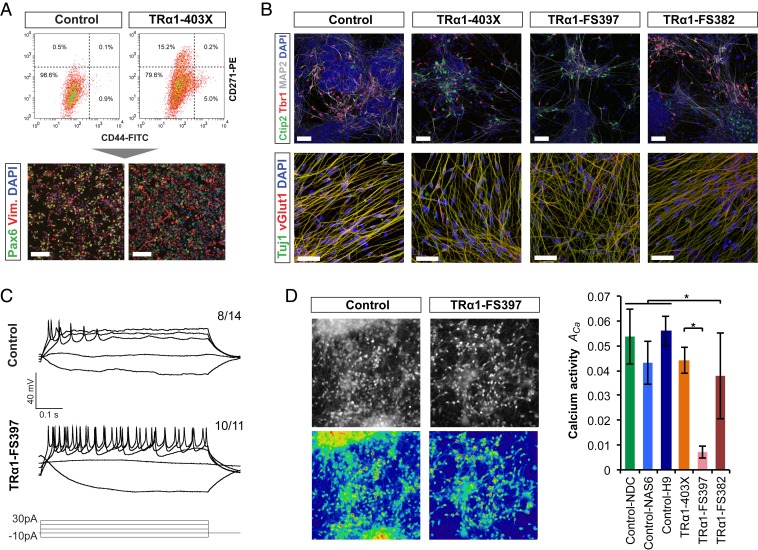
FACS-enriched *THRA* mutation-containing cortical progenitor cells generate functional neuronal networks. (*A*) Representative FACS results for WT and *THRA* mutation-containing cells at day 18. Immunocytochemistry confirmed the cortical identity of FACS- selected progenitor cells. (Scale bars: 50 μm.) (*B*) Neurons produced by FACS-purified progenitor cells expressed the cortical layer-specific transcription factors TBR1 (layer VI) and CTIP2 (layer V), as well as the vesicular glutamate transporter vGlut1, at day 40. (Scale bars: 50 μm.) (*C*) Representative traces showing action potential firing in response to stepwise current injection in *THRA* mutation-containing and control neurons. Numbers indicate the proportion of cells that fired action potentials. (*D*) Calcium indicator Oregon Green BAPTA was used as a proxy for action potential firing to measure spontaneous neuronal activity. Fluorescence images show control and *THRA* mutant cultures after loading. Heat maps highlight different levels of activity across the cultures. Total calcium activity across the field of view, $A_{Ca}$, was quantified based on 3 to 6 videos from each cell line (403X and FS382, *n* = 6 samples from 2 independent inductions; all others, *n* = 3 samples from 1 induction; *P < 0.05, Student’s *t* test). Error bars indicate SEM.

To compare neural development from *THRA* mutation-containing versus control cortical progenitors while controlling for differences in induction efficiency, cultures were enriched for neural stem cells by FACS at day 16 after neural induction. Based on an established neural stem cell signature (28), the CD271^−^CD44^−^ population was isolated and propagated (Fig. 4*A*). CD271 is expressed in neural crest precursors (29, 30), which are occasionally observed as a contaminant in control cortical inductions in vitro and identified based on their characteristic morphology as more prominent in *THRA* mutant inductions. Negative selection for CD44, a pluripotent stem cell marker (28), eliminated cells that had failed induction to neural stem cell fate. Depending on induction efficiency, approximately 50% to 80% of induced *THRA* mutation-containing cells were FACS-selected for propagation (Fig. 4*A*). FACS-enriched cultures expressed PAX6 and the intermediate filament protein vimentin, both of which are neural progenitor cell markers (Fig. 4*A* and *SI Appendix*, Fig. S2*F*).

Over time, FACS-sorted progenitors gave rise to neurons expressing the transcription factors TBR1 and CTIP2, expressed by deep cortical layer neurons (Fig. 4*B*). Most neurons also showed punctate vesicular glutamate transporter (VGLUT1) expression, indicating their glutamatergic identity (Fig. 4*B*). Overall, FACS successfully enriched for cortical neural progenitors derived from *THRA* mutation-containing iPSCs, which proceeded to generate cortical excitatory neurons over time in vitro.

To assess neuronal function, 60-d-old *THRA* mutation-containing and control cultures were analyzed by single-cell electrophysiology. The *THRA* mutation-containing neurons fired action potentials normally in response to stepwise current injections (Fig. 4*C*) and displayed sodium and potassium currents in response to voltage stimulation (*SI Appendix*, Fig. S3*A*). Spontaneous miniature excitatory postsynaptic currents were observed in a similar proportion of *THRA* mutation-containing and control neurons (*SI Appendix*, Fig. S3*A*). The *THRA* mutation-containing neurons appeared more mature than controls, with a greater proportion of cells firing action potentials (10/11 in FS397 vs. 8/14 in H9; *P* < 0.1, 2-proportion Z-test), more action potentials fired per burst (mean, 7.3 ± 2.5 in FS397 vs. 1.6 ± 1.0 in H9; *P* < 0.05, Student’s *t* test), larger mean peak sodium currents (mean, −398 ± 66 in FS397 vs. −225 ± 48 in H9; *P* < 0.05, Student’s *t* test), and a lower average membrane potential (−54 ± 2 mV in FS397 vs. −48 ± 3 mV in H9; *P* = 0.12, Student’s *t* test). These differences might represent variations in individual neuronal properties but could also be due to earlier production of neurons in *THRA* mutation-containing cultures.

By day 50 postinduction, both control and *THRA* mutation-containing cells developed spontaneous neuronal network activity, visualized by calcium imaging (Fig. 4*D* and *SI Appendix*, Fig. S3*B*). Spontaneous activity was blocked by the sodium channel blocker tetrodotoxin and substantially reduced by the AMPA receptor antagonist CNQX, indicating that a significant element of the observed spontaneous neuronal activity is mediated by excitatory synaptic transmission (*SI Appendix*, Fig. S3*C*).

### Compromised Cortical Progenitor Cell Dynamics in *THRA* Mutation-Containing Cells.

**Fig. 5.**
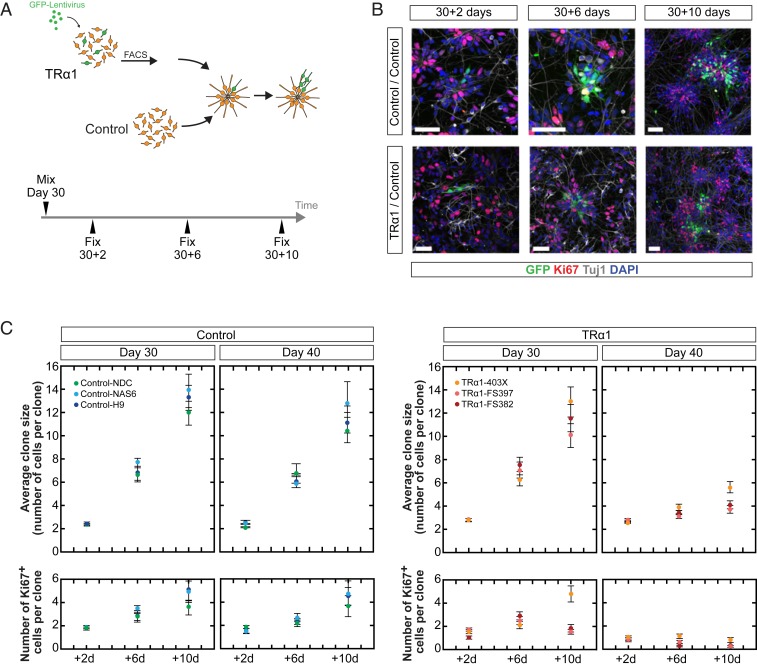
Clonal lineage analysis reveals marked differences between *THRA* mutation-containing and control progenitor cell dynamics. (*A*) Schematic of the experimental protocol. GFP-labeled cortical progenitor cells were enriched for by FACS and mixed with WT cultures at day 30 or 40. Cultures were fixed for analysis after 2, 6, or 10 d of incubation. (*B*) Representative images of *THRA* mutant and control clones derived from a single progenitor cell after 2, 6, and 10 d, immunostained for Ki67 (progenitors) and βIII-tubulin (neurons). (Scale bars: 50 μm.) (*C*) Average clone size and average number of Ki67^+^ cells per clone over time; each data point represents the average of *n* = 50 to 72 clones. Error bars indicate SEM.

The microencephaly and reduced white matter tract density seen in patients with RTHα suggest that structural differences in the cerebral cortex might contribute to their neurocognitive phenotype. With human cortex comprising columns of clonally related excitatory neurons preferentially synapsing with one another (31), the clonal output of a cortical progenitor cell is a major contributor to cortical size, architecture, and function. To determine whether defects in early cortical development could contribute to the neurologic phenotypes of patients, we undertook single-cell clonal lineage analysis of cortical progenitors, comparing cell dynamics and clonal output of *THRA* mutation-containing cells vs. control cells. A small proportion (≤1%) of *THRA* mutation-containing or control cortical progenitor cells, infected with a GFP-expressing replication-incompetent lentivirus, were mixed with unlabeled control cultures at day 30 and day 40 after cortical induction. Their progeny was then traced over time, enabling the delineation of cell-intrinsic differences between *THRA* mutation-containing and control progenitor cells (Fig. 5*A*).

The size and composition of clones (2 or more cells) derived from labeled progenitors were analyzed at 2, 6, and 10 d after mixing. The majority of labeled cells expressed either Ki67 or the neuron-specific marker βIII-tubulin, indicating that clones consisted of cycling progenitors and neurons (Fig. 5*B*). Across the 3 *THRA* mutation-containing and 3 control cell lines (human iPSC lines NDC and NAS6 and human embryonic stem cell line H9), the average size of clones increased progressively over the 10-d period after labeling (Fig. 5*C*). Average clone sizes were similar in control cortical progenitor cells labeled at either day 30 or day 40, whereas the size of *THRA* mutation-containing clones labeled at day 40 was markedly reduced.

Analyzing clone composition, the average number of progenitor cells per clone (as assessed by Ki67 expression) was increased in control cultures at day 30 and 40 (Fig. 5*C*). In *THRA* mutation-containing cultures, progenitor cell numbers increased only slightly at day 30 and decreased at day 40, suggesting a differing pattern of cell division with earlier cell cycle exit of *THRA* mutation-containing progenitors. Thus, depletion of the THRA mutation-containing progenitor pool leads to premature neuron production and a decrease in clonal output over time. By live imaging of cells over a 48-h period, we confirmed that these differences were not due to altered cell cycle kinetics or increased cell death (*SI Appendix*, Fig. S4). Furthermore, the majority of KI67^−^ cells were TUJ1^+^ in both control and *THRA* mutation-containing cell cultures (Fig. 5*B*); thus, any difference in the proportion of progenitors entering quiescence, if present, is likely negligible.

### Premature Cell Cycle Exit of *THRA* Mutation-Containing Cortical Progenitor Cells.

**Fig. 6.**
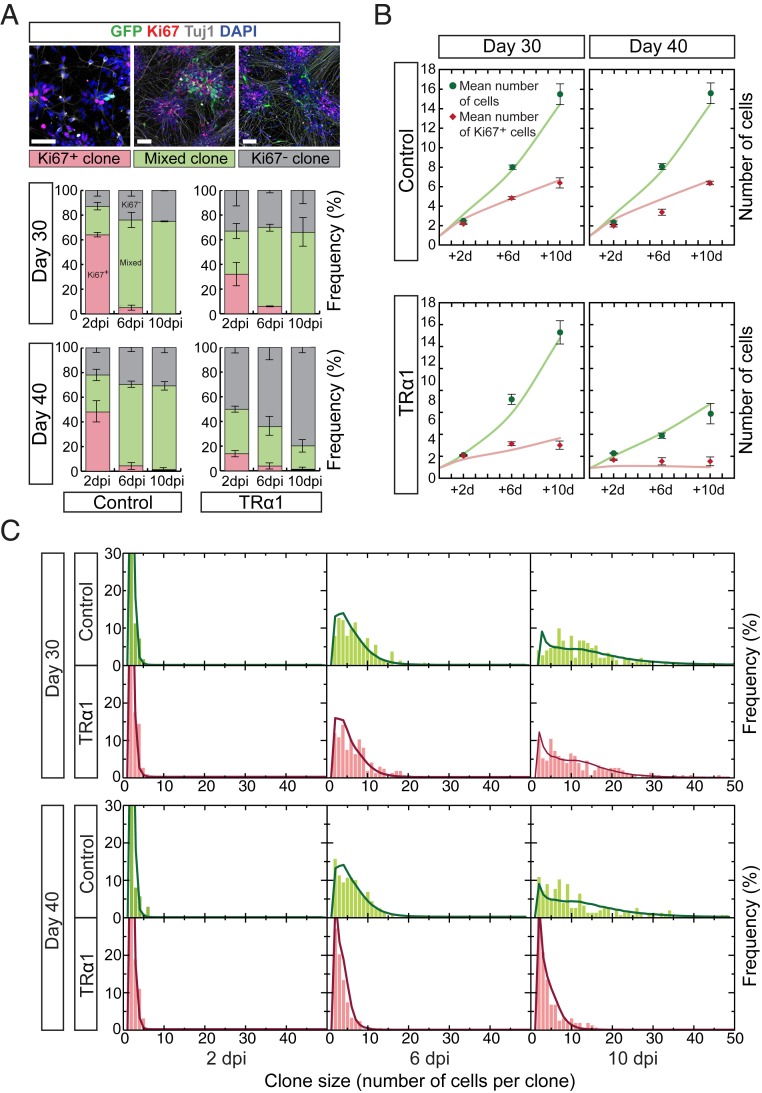
Clonal dynamics are consistent with a simple model of reduced progenitor output in *THRA* mutants. (*A*) The frequency of clones consisting of only Ki67^+^ cells (rose), only Ki67^−^ cells (gray), or both (green) at the different time points (days postmixing, [dpm]). (Scale bar: 50 μm.) (*B*) Average clone sizes of persisting clones, with model fit (green line), and average number of Ki67^+^ cells with the model prediction using the best-fitting parameters (red line). (*C*) Total clone size distributions and model predictions (lines); each histogram represents *n* = 152 to 176 clones. In *A* and *B*, data points represent the average of 3 control and 3 *THRA* mutant lines; error bars indicate SEM.

To further quantitate the division pattern of progenitor cells, fully differentiated clones were distinguished from persisting clones containing at least 1 progenitor cell (Fig. 6*A*). The proportion of persisting clones decreased more rapidly in *THRA* mutation-containing than control cultures (Fig. 6*A*). At the same time, the average size of persisting clones increased near-exponentially in controls but more linearly in *THRA* mutant cells at day 40 (Fig. 6*B*), suggesting that a higher proportion of control progenitor cells are self-renewing symmetrically, whereas *THRA* mutation-containing progenitor cells have transitioned to a more asymmetric, neurogenic division pattern.

To test this, we used a computational model of human cortical development in which self-renewing radial glia (RGs) produce intermediate progenitor cells (IPCs), which generate neurons through asymmetric or terminal divisions (*SI Appendix*, *Supplementary Methods* and Fig. S5). Based on the findings of a recent in vivo genetic labeling study of cortical neurogenesis in mouse, we presumed that RGs transit from a phase of symmetrical proliferation to a neurogenic phase, in which they divide asymmetrically to give rise to IPCs with variable but limited neurogenic potential (32). We found that this model could largely account for differences in the clonal behavior of *THRA* mutation-containing vs. control cells, including the distribution of clone size and composition, under the single assumption that *THRA* mutation-containing RGs differentiated into IPCs at a higher rate compared with control RGs (Fig. 6 *B* and *C*). Therefore, the clonal lineage data and the computational model together demonstrate that *THRA* mutation-containing progenitor cells switch to neurogenesis earlier than control cells, which continue proliferating.

During human neurogenesis, the deepest neuronal layers of the cerebral cortex emerge first, whereas neurons of the upper layers appear subsequently and migrate away from the progenitor cell zone to their final positions. Therefore, expression of TBR1, which is high in layer VI neurons in the developing cortex, is thought to precede expression of CTIP2, which is low in layer VI neurons but high in layer V neurons (33). Quantification of CTIP2 and TBR1 expression in *THRA* mutation-containing and control cells at day 40 showed significantly more TBR1^+^ and CTIP2^+^ cells in mutant compared with control cell cultures, providing independent evidence for premature neuronal differentiation of *THRA* mutation-containing progenitor cells (*SI Appendix*, Fig. S6).

### Cell Polarity and Rosette Self-Assembly Defects in *THRA* Mutation-Containing Cortical Progenitor Cells.

**Fig. 7.**
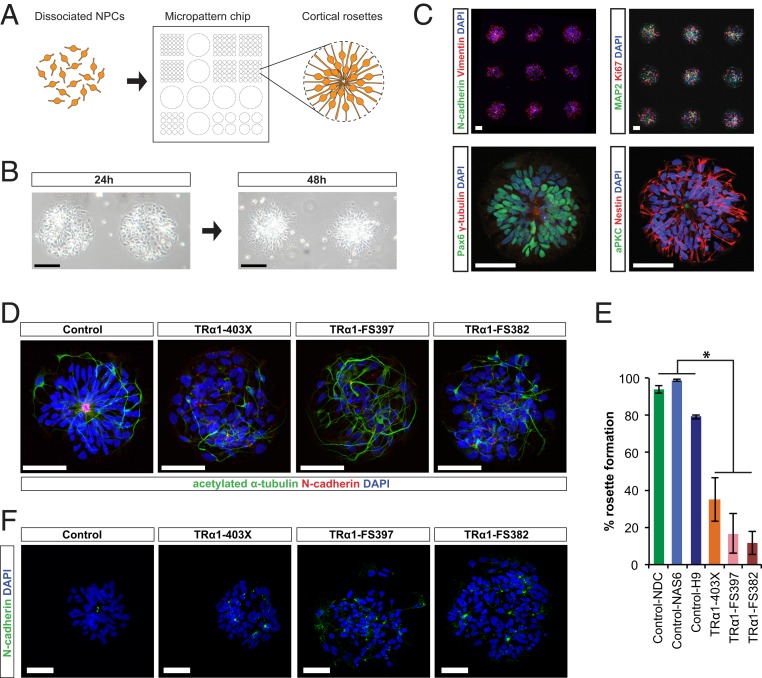
Cortical rosette self-organized assembly is impaired in *THRA* mutant cultures. (*A*) Schematic of the experimental protocol. Dissociated NPCs were plated onto micropatterned chips containing fields of diameter of 140 μm and cultured for several days. (*B*) Phase-contrast images of individual micropatterned fields at 24 h and 48 h after plating (control-H9 cells). (Scale bar: 100 μm.) (*C*) Immunostaining of rosettes fixed at 48 h after plating (control cells). Since RGs in vitro are connected by adherens junctions (AJs) at their apical ends (22), expression of the AJ component N-cadherin (*Top Left*) was used to visualize rosette centers. Rosettes contained Ki67^+^ cycling cells, and MAP2^+^ neurons were frequently observed at the periphery (*Top Right*). The centrosome protein γ-tubulin and the apically expressed atypical protein kinase C were localized to rosette centers (*Bottom*), demonstrating the apical-basal polarity of Pax6^+^ and Nestin^+^ progenitor cells. (Scale bars: 50 μm.) (*D*) Representative images of *THRA* mutant and control progenitors plated onto micropatterned chips. N-cadherin (red) was used to visualize rosette centers. (Scale bars: 50 μm.) (*E*) Bar chart summarizing the proportion of fields of diameter of 140 μm that were occupied by a single rosette (*n* = 405 for TD, 267 for CK, 187 for KB, 72 for NDC, 279 for NAS6, and 165 for H9, from 2 independent inductions per cell line; *P < 0.001, Student’s *t* test comparing the 6 *THRA* mutant vs. 6 control inductions). (*F*) Representative images of *THRA* mutant and control progenitors plated onto micropatterned chips. N-cadherin expression (green) is localized to rosette centers in control but not in *THRA* mutant cultures. (Scale bars: 50 μm.)

Previous studies have shown that cortical progenitor cell polarity and attachment at the ventricular surface maintain progenitor identity and proliferation in the mouse cortex (34), consistent with foregoing our gene expression data. The functional units of corticogenesis in the in vitro system are rosette-like arrangements of progenitor cells, which self-assemble and recreate aspects of the in vivo niche (22). We observed that following dissociation, *THRA* mutation-containing progenitors self-organized into rosettes less readily than controls. To quantify the efficiency of rosette formation, progenitor cells were plated at a defined density onto micropatterned chips that only allow cell adhesion in circular areas of different diameters (Fig. 7*A*).

In control lines, rosettes formed efficiently within 48 h after plating, with areas of 140 μm diameter predominantly hosting a single rosette (Fig. 7*B*). The organization of cell types within rosettes showed clear apical-basal polarity (Fig. 7*C*). In contrast, rosette formation was substantially impaired in *THRA* mutation-containing cell cultures (Fig. 7*D*). Treatment of control cultures with the competitive TR antagonist 1-850 (35) decreased rosette formation efficiency and proliferation, indicating that aberrant TH signaling likely contributes to impaired rosette formation (*SI Appendix*, Fig. S7*A*).

The mechanisms underlying neural rosette formation in vitro are largely enigmatic (36). Live imaging of control cultures for up to 72 h after plating found no evidence for directional movement of cells, suggesting that cells do not respond to chemotactic signals (*SI Appendix*, Fig. S7*B*). To test which factors are required for rosette formation, we developed a computational model in which cells move randomly and form attachments at their apical ends when in close proximity. Both the number of rosettes and the distribution of rosette centers observed in vitro were statistically reproduced in silico, indicating that migration and apical attachment are sufficient for rosette formation (*SI Appendix*, Fig. S7*C*). As migration defects were not apparent from the live imaging data (*SI Appendix*, Fig. S7*B*), impaired apical attachments may be limiting rosette formation efficiency in *THRA* mutation-containing cultures.

In support of this notion, N-cadherin was highly localized to the apical end of progenitor cells in control rosettes but expressed along the cell membrane in *THRA* mutation-containing cells, suggesting impaired adherens junction formation (Fig. 7 *D* and *E*), consistent with the down-regulation of protocadherins observed on RNA-seq. Apical expression of the polarity complex protein PKCζ and the centrosome-associated γ-tubulin was observed in control progenitor cells but not in *THRA* mutation-containing cells (*SI Appendix*, Fig. S7*D*). Thus, defects in progenitor cell polarity and spatial organization may contribute to their premature cell cycle exit.

## Discussion

Consistent with the known critical role of TH in brain development, we have documented neurocognitive deficits (e.g., reduced nonverbal IQ, motor incoordination, impaired visual motor integration) in patients with RTHα, with some deficits (e.g., motor incoordination, poor finger/hand dexterity) resembling consequences of untreated congenital hypothyroidism. Structural abnormalities include reduction in cerebellar volume, cortical microencephaly in adult cases, and decreased white matter tract density. The neurocognitive, structural, and functional deficits observed in patients with RTHα suggest abnormalities in several brain regions, including the axis from the cerebellum to the neostriatum and the cerebral cortex. Here we focused on the effects of mutant TRα1 on cerebral cortex development and function, using cortical neural progenitor cells derived from *THRA* mutation-containing iPSCs from these patients. While our experimental model effectively recapitulates defined aspects of corticogenesis in vitro, it does not capture other brain developmental processes, such as myelination and cerebellar development, that may also be impaired in patients and should be elucidated in future studies.

Our cortical differentiation protocol revealed that *THRA* mutation-containing cortical progenitor cells differentiate prematurely in vitro, with attendant depletion of the progenitor cell pool reducing clonal output, which in vivo would result in microencephaly. Quantitative analysis of lineage tracing data confirmed that the fate choices of *THRA* mutation-containing cortical progenitor cells are biased toward neuronal differentiation as opposed to the continued self-renewal observed in control cells. As single-labeled *THRA* mutation-containing progenitor cells were studied in WT background cultures, such alterations in clonal dynamics likely reflect distinct cell-autonomous fate behaviors.

RNA-seq of early cortical progenitor cells indicated down-regulated expression of genes involved in cell polarity or adherence in *THRA* mutation-containing cells. These included several protocadherins whose cellular functions are not fully understood but may act as adhesion molecules on their own or in association with other cadherins (37). *THRA* mutation-containing cortical progenitor cells also showed disordered expression of N-cadherin, a well-described classical cadherin, and defective self-organization into rosettes on micropatterned chips, which may be explained by reduced apical cell–cell adhesion based on our computational model. Taken together, these results indicate that apical adherens junction formation is impaired in *THRA* mutation-containing cortical progenitor cells. In rodent studies, ventricular attachment through adherens junctions is required for apical-basal polarity, spatial organization, and self-renewal of cortical RGs (34, 38, 39), and there is emerging evidence indicating that protocadherins play a role in these processes (40, 41).

In the developing cerebral cortex, neural progenitor cell interactions through adherens junctions have been shown to constitute a self-supportive niche maintaining progenitor cell proliferation and inhibiting differentiation (42). Thus, defects in cell polarity and attachment to neighboring stem cells may mediate the premature cell cycle exit observed in *THRA* mutation-containing cortical progenitor cells. Our findings also suggest that some cell-intrinsic consequences of mutant TRα1 dysfunction may become apparent only through interactions with the local niche. We note, however, that our in vitro cortical differentiation protocol generates a reduced model of the actual human cortex, lacking, for example, vasculature and surrounding supportive tissues, as well as nonneuronal cells, such as microglia. Neural progenitor cells in vivo reside in a distinct 3D architecture with axial patterning signals and neurophysiological activity not replicated in vitro. Thus, our in vitro results on the structure and organization of neural progenitor cell rosettes might not directly translate to the in vivo situation. Future studies may establish the functional hierarchy of determinants of cortical progenitor cell identity in vivo, to further delineate the mechanisms through which mutant TRα1 affects neurogenesis. It will also be important in future studies to validate the modeling presented here that predicts a change in intermediate progenitor cell dynamics.

Overall, we suggest that abnormal proliferation and adhesion of *THRA* mutation-containing cortical neural progenitors forms the basis, at least in part, of structural and functional changes culminating in neurocognitive deficits seen in patients with RTHα. We note that our lineage-tracing experiments and RNA-seq data cannot resolve whether the observed differences in gene expression and cell fate behavior are due to transcriptional changes within a comparable population of neural progenitor cells, changes in the cellular composition of the progenitor cell populations, or a combination of these 2 possibilities. As our understanding of human corticogenesis is still evolving, with the cell states and cell types involved not yet definitively identified, this distinction is at present semantic and does not affect the conclusions of our study.

Reduced cortical progenitor output and impaired cortical architecture have been observed in human patients and mouse models of several other neurodevelopmental disorders, including Down syndrome, schizophrenia, and autism spectrum disorders (43–46). A number of genes differentially expressed in *THRA* mutation-containing cells, including the cell surface dystroglycan receptor *NRXN1* (neurexin) and the transmembrane protein *NPTN* (neuroplastin), have also been implicated in other neurodevelopmental disorders (26, 27). Since normal cortical progenitor self-renewal requires the orchestration of a range of cell-intrinsic and environmental factors, including transcription factors determining cortical stem cell identity and genes regulating cell attachment, proliferation, and movement, as well as cytoskeletal organization and polarity (34), a range of gene defects may converge onto similar abnormal neurodevelopmental phenotypes. Specifically, our observations suggest that impaired cell adhesion, leading to defective cortical size and architecture, may ultimately result in neurocognitive deficits across a range of genetic disorders.

In addition to mouse models, novel human iPSC-derived in vitro systems are being increasingly used to study neurodevelopmental conditions, hitherto focusing on neuronal maturation and network function (47–51). Extending these approaches, this study describes a method of assessing the proliferative potential and fate of neural stem cells in vitro. Through quantitative analysis of lineage tracing data, even minor differences in clonal dynamics can be resolved. In addition, we have developed a quantitative assay for the self-organizing capacity of neural stem cells. Together, these approaches provide an experimental platform to investigate the function of cortical progenitor cells with differing genetic backgrounds, study neural progenitor cell dynamics during normal and pathological development, and ultimately screen for agents to ameliorate defective neurogenesis.

## Materials and Methods

The methodology used in this study is described in more detail in *SI Appendix*.

### Neuroimaging.

Whole-brain MRI, DTI, and proton MRS were performed at Great Ormond Street Hospital, London and compared with data obtained from healthy age-matched controls. All clinical investigations, including neuroimaging and derivation of iPSCs, were undertaken as part of a protocol approved by our Research Ethics Committee (Cambridgeshire; LREC 98/154) or were clinically indicated and were performed with previous written informed consent of patients and/or parents.

### Neural Differentiation of iPSCs.

Neural induction of human control PSCs and *THRA* mutant iPSCs was performed as described previously (21). For FACS sorting, 10^6^ cells per sample were collected and stained with fluorochrome-conjugated antibodies. Total RNA from cortical cultures was isolated using TRIzol (Sigma-Aldrich), and semiquantitative RT-PCR was performed using primers against *FOXG1*, *PAX6*, and *GAPDH*. For Western blot analysis, protein was extracted from overnight-frozen cell pellets, and the insoluble fraction was removed by centrifugation. Band intensity was measured using Image Studio software (Li-COR) and normalized to β-actin. Immunofluorescence staining was performed following fixation with 4% paraformaldehyde.

### Electrophysiology and Calcium Imaging.

For electrophysiological recordings, cortical neurons were incubated with artificial cerebral spinal fluid and subjected to step depolarizations or stepwise current injections. Recordings were made using a Multiclamp 700 A amplifier (Molecular Devices). For calcium imaging, cells were incubated with calcium indicator Oregon Green 488 BAPTA, and calcium activity was recorded on a Deltavision fluorescence microscope with an EMCCD camera (Applied Precision).

### Clonal Lineage Analysis.

For clonal lineage analysis, third-generation replication-incompetent lentivirus was produced by calcium phosphate transfection of HEK293T cells, using pBOP-GFP plasmids combined with packaging plasmids pRSV-Rev, pMDLg/pRRE, and pMD2.G. Cortical progenitor cells were infected at high titer before isolation of the CD271^−^CD44^−^ population by FACS. At day 30 and day 40, sorted progenitors were mixed with unlabeled control cultures. These mixed cultures were fixed and immunostained at 2, 6, and 10 d after plating.

### Micropattern Chip Cultures.

For micropattern chip cultures, 10^6^ cells were plated onto laminin-coated CYTOOchips and cultured for 1 to 7 d. For live imaging, tissue culture dishes containing cells in N2B27 medium were imaged in a BioStation CT (Nikon) at 37 °C with 7% CO_2_.

### RNA-Seq.

For RNA-seq, total RNA was extracted from 3 control lines and 3 *THRA* mutant lines at day 12 of in vitro cortical induction. Libraries were prepared using the Illumina TruSeq Stranded Total RNA sample preparation kit and single-end sequenced on an Illumina HiSeq 1500 system. Gene expression profiles were clustered using GeneE software, based on Pearson’s correlation. GO analysis was performed using the PANTHER database (www.panther.org).

## Supplementary Material

Supplementary File

## References

[1] Bath S. C., Steer C. D., Golding J., Emmett P., Rayman M. P., Effect of inadequate iodine status in UK pregnant women on cognitive outcomes in their children: Results from the Avon Longitudinal Study of Parents and Children (ALSPAC). Lancet 382, 331–337 (2013). 23706508 10.1016/S0140-6736(13)60436-5

[2] Mohan V., Maternal thyroid hormone deficiency affects the fetal neocorticogenesis by reducing the proliferating pool, rate of neurogenesis and indirect neurogenesis. Exp. Neurol. 237, 477–488 (2012). 22892247 10.1016/j.expneurol.2012.07.019

[3] Pathak A., Sinha R. A., Mohan V., Mitra K., Godbole M. M., Maternal thyroid hormone before the onset of fetal thyroid function regulates reelin and downstream signaling cascade affecting neocortical neuronal migration. Cereb. Cortex 21, 11–21 (2011). 20368265 10.1093/cercor/bhq052

[4] Lavado-Autric R., Early maternal hypothyroxinemia alters histogenesis and cerebral cortex cytoarchitecture of the progeny. J. Clin. Invest. 111, 1073–1082 (2003). 12671057 10.1172/JCI16262 PMC152582

[5] Ausó E., A moderate and transient deficiency of maternal thyroid function at the beginning of fetal neocorticogenesis alters neuronal migration. Endocrinology 145, 4037–4047 (2004). 15087434 10.1210/en.2004-0274

[6] Moog N. K., Influence of maternal thyroid hormones during gestation on fetal brain development. Neuroscience 342, 68–100 (2017). 26434624 10.1016/j.neuroscience.2015.09.070 PMC4819012

[7] Horn S., Heuer H., Thyroid hormone action during brain development: More questions than answers. Mol. Cell. Endocrinol. 315, 19–26 (2010). 19765631 10.1016/j.mce.2009.09.008

[8] Bernal J., Thyroid hormone receptors in brain development and function. Nat. Clin. Pract. Endocrinol. Metab. 3, 249–259 (2007). 17315033 10.1038/ncpendmet0424

[9] Vaquerizas J. M., Kummerfeld S. K., Teichmann S. A., Luscombe N. M., A census of human transcription factors: Function, expression and evolution. Nat. Rev. Genet. 10, 252–263 (2009). 19274049 10.1038/nrg2538

[10] Bradley D. J., Towle H. C., Young W. S. 3rd, Spatial and temporal expression of alpha- and beta-thyroid hormone receptor mRNAs, including the beta 2-subtype, in the developing mammalian nervous system. J. Neurosci. 12, 2288–2302 (1992). 1607941 10.1523/JNEUROSCI.12-06-02288.1992 PMC6575910

[11] Astapova I., The nuclear corepressor, NCoR, regulates thyroid hormone action in vivo. Proc. Natl. Acad. Sci. U.S.A. 105, 19544–19549 (2008). 19052228 10.1073/pnas.0804604105 PMC2614797

[12] Ito M., Yuan C. X., Okano H. J., Darnell R. B., Roeder R. G., Involvement of the TRAP220 component of the TRAP/SMCC coactivator complex in embryonic development and thyroid hormone action. Mol. Cell 5, 683–693 (2000). 10882104 10.1016/s1097-2765(00)80247-6

[13] Bochukova E., A mutation in the thyroid hormone receptor alpha gene. N. Engl. J. Med. 366, 243–249 (2012). 22168587 10.1056/NEJMoa1110296

[14] van Mullem A., Clinical phenotype and mutant TRα1. N. Engl. J. Med. 366, 1451–1453 (2012). 10.1056/NEJMc1113940 22494134

[15] Moran C., Resistance to thyroid hormone caused by a mutation in thyroid hormone receptor (TR)α1 and TRα2: Clinical, biochemical, and genetic analyses of three related patients. Lancet Diabetes Endocrinol. 2, 619–626 (2014). 24969835 10.1016/S2213-8587(14)70111-1 PMC5989926

[16] Moran C., An adult female with resistance to thyroid hormone mediated by defective thyroid hormone receptor α. J. Clin. Endocrinol. Metab. 98, 4254–4261 (2013). 23940126 10.1210/jc.2013-2215

[17] Espiard S., A novel mutation in THRA gene associated with an atypical phenotype of resistance to thyroid hormone. J. Clin. Endocrinol. Metab. 100, 2841–2848 (2015). 26037512 10.1210/jc.2015-1120

[18] Tylki-Szymańska A., Thyroid hormone resistance syndrome due to mutations in the thyroid hormone receptor α gene (THRA). J. Med. Genet. 52, 312–316 (2015). 25670821 10.1136/jmedgenet-2014-102936

[19] Fauquier T., Severe impairment of cerebellum development in mice expressing a dominant-negative mutation inactivating thyroid hormone receptor alpha1 isoform. Dev. Biol. 356, 350–358 (2011). 21621530 10.1016/j.ydbio.2011.05.657

[20] Moran C., Chatterjee K., Resistance to thyroid hormone α-emerging definition of a disorder of thyroid hormone action. J. Clin. Endocrinol. Metab. 101, 2636–2639 (2016). 27381958 10.1210/jc.2016-2317

[21] Shi Y., Kirwan P., Livesey F. J., Directed differentiation of human pluripotent stem cells to cerebral cortex neurons and neural networks. Nat. Protoc. 7, 1836–1846 (2012). 22976355 10.1038/nprot.2012.116

[22] Shi Y., Kirwan P., Smith J., Robinson H. P. C., Livesey F. J., Human cerebral cortex development from pluripotent stem cells to functional excitatory synapses. Nat. Neurosci. 15, 477–486 (2012). 22306606 10.1038/nn.3041 PMC3882590

[23] Park I.-H., Disease-specific induced pluripotent stem cells. Cell 134, 877–886 (2008). 18691744 10.1016/j.cell.2008.07.041 PMC2633781

[24] Johnson M. B., Single-cell analysis reveals transcriptional heterogeneity of neural progenitors in human cortex. Nat. Neurosci. 18, 637–646 (2015). 25734491 10.1038/nn.3980 PMC5568903

[25] Chou S. J., Perez-Garcia C. G., Kroll T. T., O’Leary D. D. M., Lhx2 specifies regional fate in Emx1 lineage of telencephalic progenitors generating cerebral cortex. Nat. Neurosci. 12, 1381–1389 (2009). 19820705 10.1038/nn.2427 PMC2897740

[26] Kim H.-G., Disruption of neurexin 1 associated with autism spectrum disorder. Am. J. Hum. Genet. 82, 199–207 (2008). 18179900 10.1016/j.ajhg.2007.09.011 PMC2253961

[27] Desrivières S.; IMAGEN Consortium, Single nucleotide polymorphism in the neuroplastin locus associates with cortical thickness and intellectual ability in adolescents. Mol. Psychiatry 20, 263–274 (2015). 24514566 10.1038/mp.2013.197 PMC4051592

[28] Yuan S. H., Cell-surface marker signatures for the isolation of neural stem cells, glia and neurons derived from human pluripotent stem cells. PLoS One 6, e17540 (2011). 21407814 10.1371/journal.pone.0017540 PMC3047583

[29] Lee G., Isolation and directed differentiation of neural crest stem cells derived from human embryonic stem cells. Nat. Biotechnol. 25, 1468–1475 (2007). 18037878 10.1038/nbt1365

[30] Morrison S. J., White P. M., Zock C., Anderson D. J., Prospective identification, isolation by flow cytometry, and in vivo self-renewal of multipotent mammalian neural crest stem cells. Cell 96, 737–749 (1999). 10089888 10.1016/s0092-8674(00)80583-8

[31] Yu Y.-C., Bultje R. S., Wang X., Shi S.-H., Specific synapses develop preferentially among sister excitatory neurons in the neocortex. Nature 458, 501–504 (2009). 19204731 10.1038/nature07722 PMC2727717

[32] Gao P., Deterministic progenitor behavior and unitary production of neurons in the neocortex. Cell 159, 775–788 (2014). 25417155 10.1016/j.cell.2014.10.027 PMC4225456

[33] Molyneaux B. J., Arlotta P., Menezes J. R. L., Macklis J. D., Neuronal subtype specification in the cerebral cortex. Nat. Rev. Neurosci. 8, 427–437 (2007). 17514196 10.1038/nrn2151

[34] Götz M., Huttner W. B., The cell biology of neurogenesis. Nat. Rev. Mol. Cell Biol. 6, 777–788 (2005). 16314867 10.1038/nrm1739

[35] Schapira M., Discovery of diverse thyroid hormone receptor antagonists by high-throughput docking. Proc. Natl. Acad. Sci. U.S.A. 100, 7354–7359 (2003). 12777627 10.1073/pnas.1131854100 PMC165879

[36] Karus M., Blaess S., Brüstle O., Self-organization of neural tissue architectures from pluripotent stem cells. J. Comp. Neurol. 522, 2831–2844 (2014). 24737617 10.1002/cne.23608

[37] Weiner J. A., Jontes J. D., Protocadherins, not prototypical: A complex tale of their interactions, expression, and functions. Front. Mol. Neurosci. 6, 4 (2013). 23515683 10.3389/fnmol.2013.00004 PMC3601302

[38] Kosodo Y., Asymmetric distribution of the apical plasma membrane during neurogenic divisions of mammalian neuroepithelial cells. EMBO J. 23, 2314–2324 (2004). 15141162 10.1038/sj.emboj.7600223 PMC419905

[39] Kadowaki M., N-cadherin mediates cortical organization in the mouse brain. Dev. Biol. 304, 22–33 (2007). 17222817 10.1016/j.ydbio.2006.12.014

[40] Compagnucci C., Characterizing PCDH19 in human induced pluripotent stem cells (iPSCs) and iPSC-derived developing neurons: Emerging role of a protein involved in controlling polarity during neurogenesis. Oncotarget 6, 26804–26813 (2015). 26450854 10.18632/oncotarget.5757 PMC4694954

[41] Zhang P., Protocadherin 11 x regulates differentiation and proliferation of neural stem cell in vitro and in vivo. J. Mol. Neurosci. 54, 199–210 (2014). 24647733 10.1007/s12031-014-0275-x

[42] Zhang J., Cortical neural precursors inhibit their own differentiation via N-cadherin maintenance of β-catenin signaling. Dev. Cell 18, 472–479 (2010). 20230753 10.1016/j.devcel.2009.12.025 PMC2865854

[43] Chakrabarti L., Galdzicki Z., Haydar T. F., Defects in embryonic neurogenesis and initial synapse formation in the forebrain of the Ts65Dn mouse model of Down syndrome. J. Neurosci. 27, 11483–11495 (2007). 17959791 10.1523/JNEUROSCI.3406-07.2007 PMC6673208

[44] Meechan D. W., Tucker E. S., Maynard T. M., LaMantia A.-S., Diminished dosage of 22q11 genes disrupts neurogenesis and cortical development in a mouse model of 22q11 deletion/DiGeorge syndrome. Proc. Natl. Acad. Sci. U.S.A. 106, 16434–16445 (2009). 19805316 10.1073/pnas.0905696106 PMC2752572

[45] Gallagher D., Ankrd11 is a chromatin regulator involved in autism that is essential for neural development. Dev. Cell 32, 31–42 (2015). 25556659 10.1016/j.devcel.2014.11.031

[46] LaMonica B. E., Lui J. H., Wang X., Kriegstein A. R., OSVZ progenitors in the human cortex: An updated perspective on neurodevelopmental disease. Curr. Opin. Neurobiol. 22, 747–753 (2012). 22487088 10.1016/j.conb.2012.03.006 PMC3402619

[47] Brennand K. J., Modelling schizophrenia using human induced pluripotent stem cells. Nature 473, 221–225 (2011). 21490598 10.1038/nature09915 PMC3392969

[48] Paşca S. P., Using iPSC-derived neurons to uncover cellular phenotypes associated with Timothy syndrome. Nat. Med. 17, 1657–1662 (2011). 22120178 10.1038/nm.2576 PMC3517299

[49] Chamberlain S. J., Induced pluripotent stem cell models of the genomic imprinting disorders Angelman and Prader-Willi syndromes. Proc. Natl. Acad. Sci. U.S.A. 107, 17668–17673 (2010). 20876107 10.1073/pnas.1004487107 PMC2955112

[50] Marchetto M. C. N., A model for neural development and treatment of Rett syndrome using human induced pluripotent stem cells. Cell 143, 527–539 (2010). 21074045 10.1016/j.cell.2010.10.016 PMC3003590

[51] Muotri A. R., L1 retrotransposition in neurons is modulated by MeCP2. Nature 468, 443–446 (2010). 21085180 10.1038/nature09544 PMC3059197

