# Supplementary material for: Mutations in thyroid hormone receptor α1 cause premature neurogenesis and progenitor cell depletion in human cortical development
Source: Proc Natl Acad Sci U S A. 2019 Oct 18;116(45):22754–63. doi: 10.1073/pnas.1908762116 (PMC6842615; doi:10.1073/pnas.1908762116)
Supplement: Supplementary File [file pnas.1908762116.sapp.pdf]

SI Appendix

Supplementary Figures

Figure S1: Cerebellar volume reduction in adult patients

MRI scans of adult patients (P3, P4) and a control subject (female, age 52 yrs), with T2-weighted axial images (top row) and T1-weighted coronal images (bottom row) showing increased CSF spaces (arrowed) around cerebellum and between folia, denoting reduced cerebellar size.

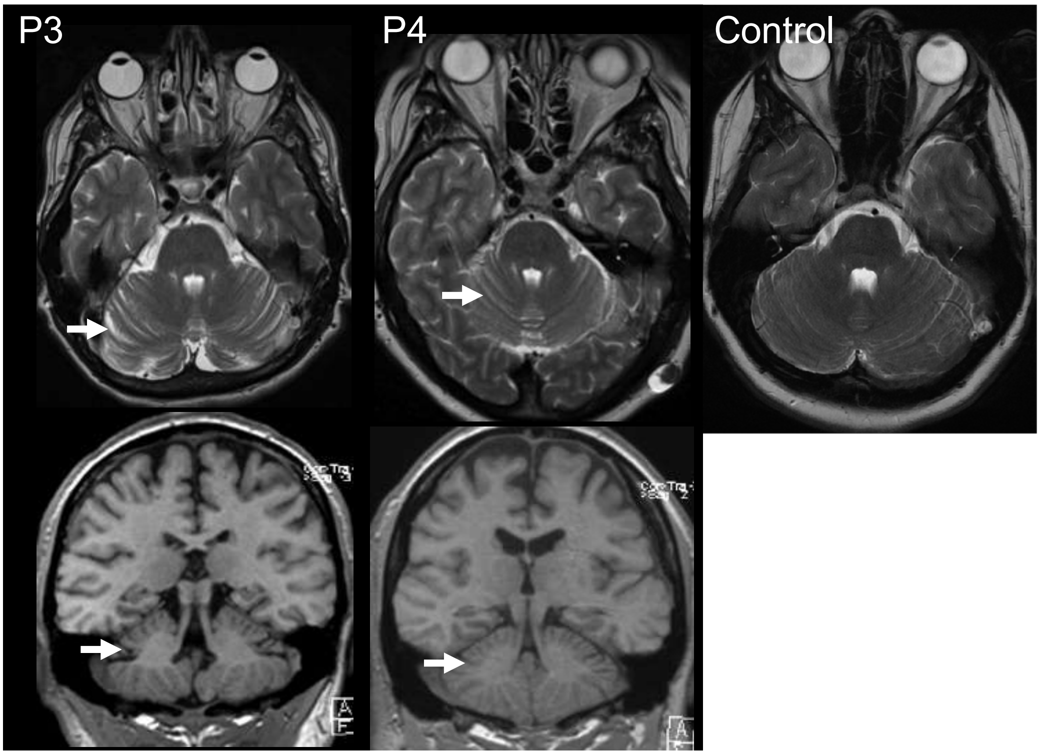

www.pnas.org/cgi/doi/10.1073/pnas.1908762116

Figure S2: *THRA* mutation-containing iPSCs can be induced to neural fate with variable efficiency

(A) Patient-derived iPSCs expressed the transcription factors Nanog, Oct4 and Sox2, as well as the membrane proteins TRA-1-60 and SSEA4, which are characteristic markers of pluripotent cells (23). The neural stem cell marker Nestin was not expressed. Scale bars, 100 μm.

(B) RT-PCR confirmed expression of Foxg1 and Pax6 in *THRA* mutation-containing and control cells (H9, NDC, NAS9). –RT, no transcriptase.

(C) Pax6 expression in TRα1 mutation-containing cells at day 12. Scale bars, 50 μm.

(D) Western blot analysis confirms TRα1 expression in control cells at D50 and D80 (green, β-actin; red, TRα1).

(E) Quantification of PAX6 and VIM expression in cultures after FACS sorting (see also Fig. 4A). Integrated PAX6 and VIM signal intensity was quantified as a fraction of integrated DAPI signal intensity. n.s., not significant ($p>0.1$, two-sided Student's t-test comparing a total of 3 control and 9 *THRA* mutant images). Error bars indicate SEM.

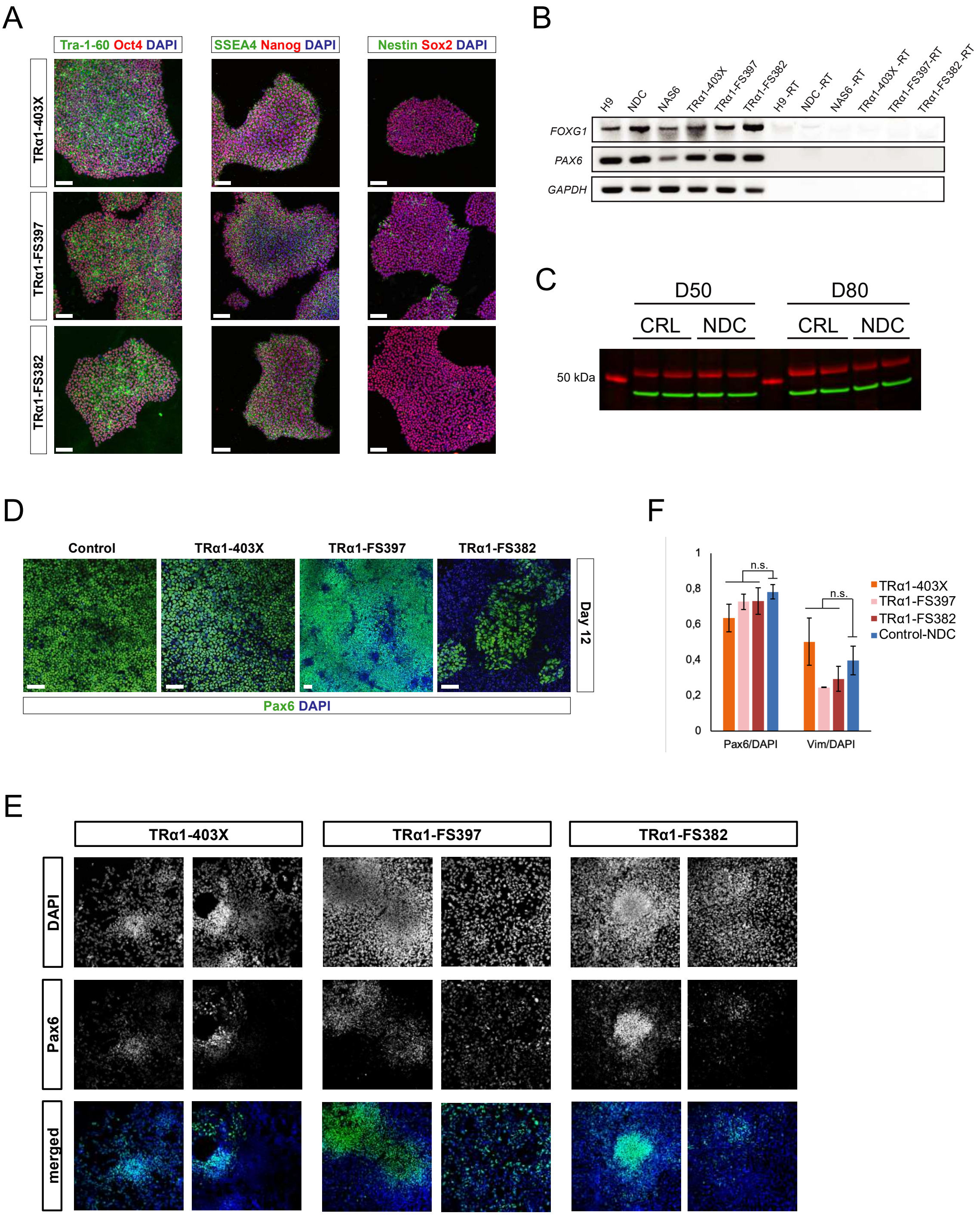
A
Tra-1-60 Oct4 DAPI
SSEA4 Nanog DAPI
Nestin Sox2 DAPI
TRα1-403X
TRα1-FS397
TRα1-FS382
B
H9
NDC
NAS6
TRα1-403X
TRα1-FS397
TRα1-FS382
H9 -RT
NDC -RT
NAS6 -RT
TRα1-403X -RT
TRα1-FS397-RT
TRα1-FS382 -RT
FOXG1
PAX6
GAPDH
C
D50
D80
CRL
NDC
CRL
NDC
50 kDa
D
Control
TRα1-403X
TRα1-FS397
TRα1-FS382
Day 12
Pax6 DAPI
F
1
0,8
0,6
0,4
0,2
0
n.s.
n.s.
TRα1-403X
TRα1-FS397
TRα1-FS382
Control-NDC
Pax6/DAPI
Vim/DAPI
E
TRα1-403X
TRα1-FS397
TRα1-FS382
DAPI
Pax6
merged

Figure S3: *THRA* mutation-containing neurons show normal electrophysiological properties and spontaneous excitatory synaptic neurotransmission

(A) Top row, representative traces showing sodium and potassium currents in response to voltage stimulation in *THRA* mutation-containing and control neurons, and quantification of the mean peak sodium current (FS397, n=14; control, n=14). * $p<0.05$ (Student's t-test). Bottom row, representative traces recorded in voltage clamp mode from *THRA* mutation-containing and control neurons held at -70 mV, and quantification of the proportion of cells that showed spontaneous activity (FS397, n=14; control, n=14).

(B) Calcium indicator Oregon Green BAPTA was used as a proxy for action potential firing to measure spontaneous neuronal activity (see Figure 3D). Representative traces show the time course of the fluorescent signal ($\Delta F/F$) observed in the ten most active cells in the field of view.

(C) Representative calcium imaging traces of *THRA* mutation-containing and control cells at day 50 before and after treatment with the sodium channel blocker tetrodotoxin (TTX) or the AMPA receptor antagonist CNQX. Spontaneous activity returned after washout (traces following washout of CNQX looked equivalent).

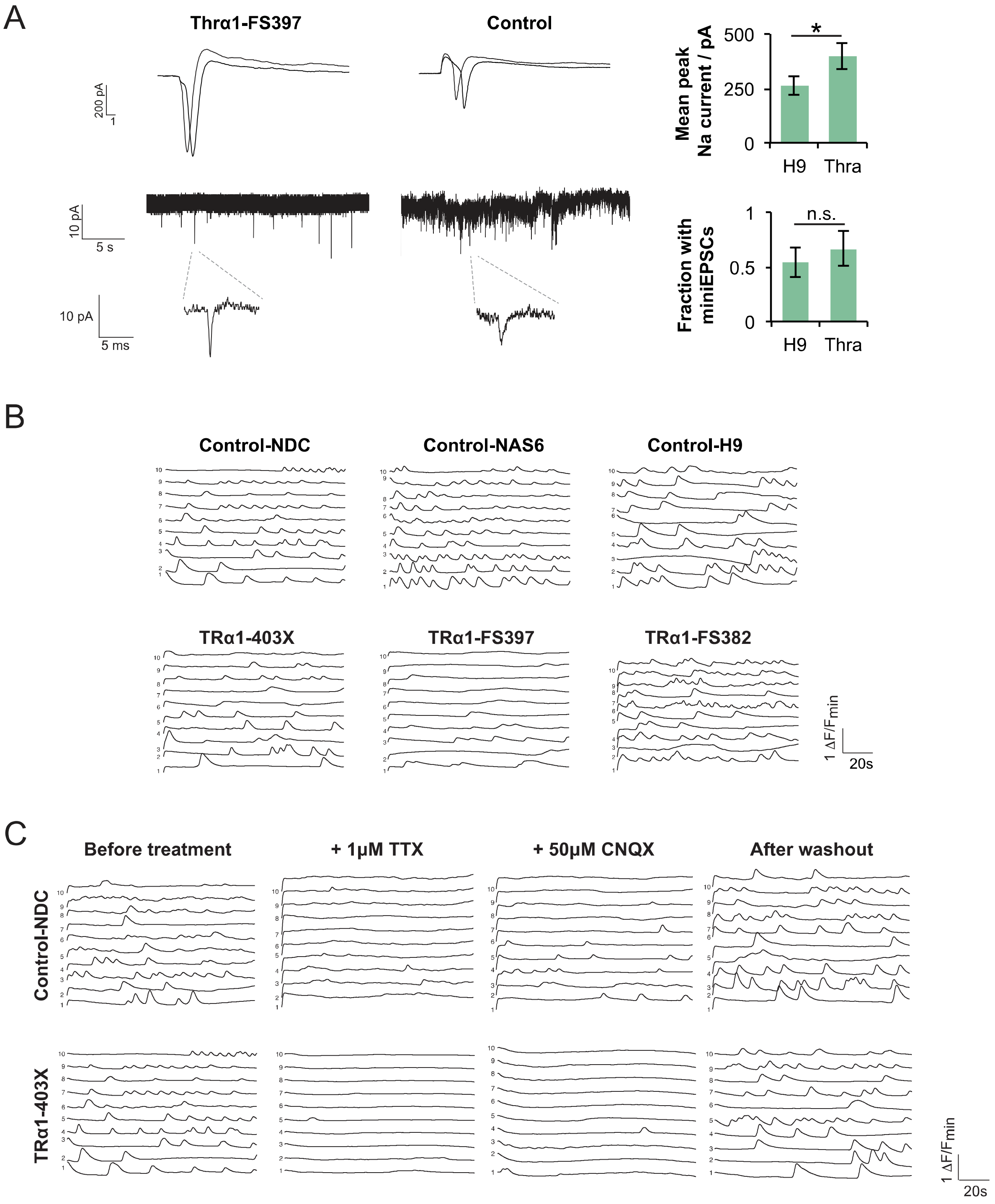

A
Thrα1-FS397
Control
200 pA
1
10 pA
5 s
10 pA
5 ms
Mean peak Na current / pA
500
250
0
H9
Thra
*
Fraction with miniEPSCs
1
0.5
0
n.s.
B
Control-NDC
Control-NAS6
Control-H9
TRα1-403X
TRα1-FS397
TRα1-FS382
1 ΔF/Fmin
20s
C
Before treatment
+ 1µM TTX
+ 50µM CNQX
After washout
Control-NDC
TRα1-403X
1 ΔF/Fmin
20s

Figure S4: Cell cycle time and cell death in *THRA* mutant and control cells

(A) Example of an apical division in which the apically anchored mother cell (green arrow) undergoes interkinetic nuclear migration and divides near the rosette centre, giving rise to two daughter cells (blue and yellow arrows) which also both retain apical processes.

(B) The distribution of cell cycle times of *THRA* mutant and control apical progenitors (n=7 for both).

(C) Average cell cycle time of *THRA* mutant and control apical progenitors. Cell cycle times were measured as the time interval between the apical divisions of a cell and its daughter cell with apical processes. Error bars indicate SEM. n.s., not significant ($p>0.01$, Student's t-test).

(D) Example of a live-imaged putative progenitor cell undergoing apoptosis.

(E) The fraction of cells that went through apoptosis over a period of 12 h. Neurons were distinguished from progenitor cells based on their characteristic morphology and location at the periphery or outside of rosettes. Error bars indicate SEM (n=100 cells were tracked for each measurement). n.s., not significant ($p>0.01$, Student's t-test).

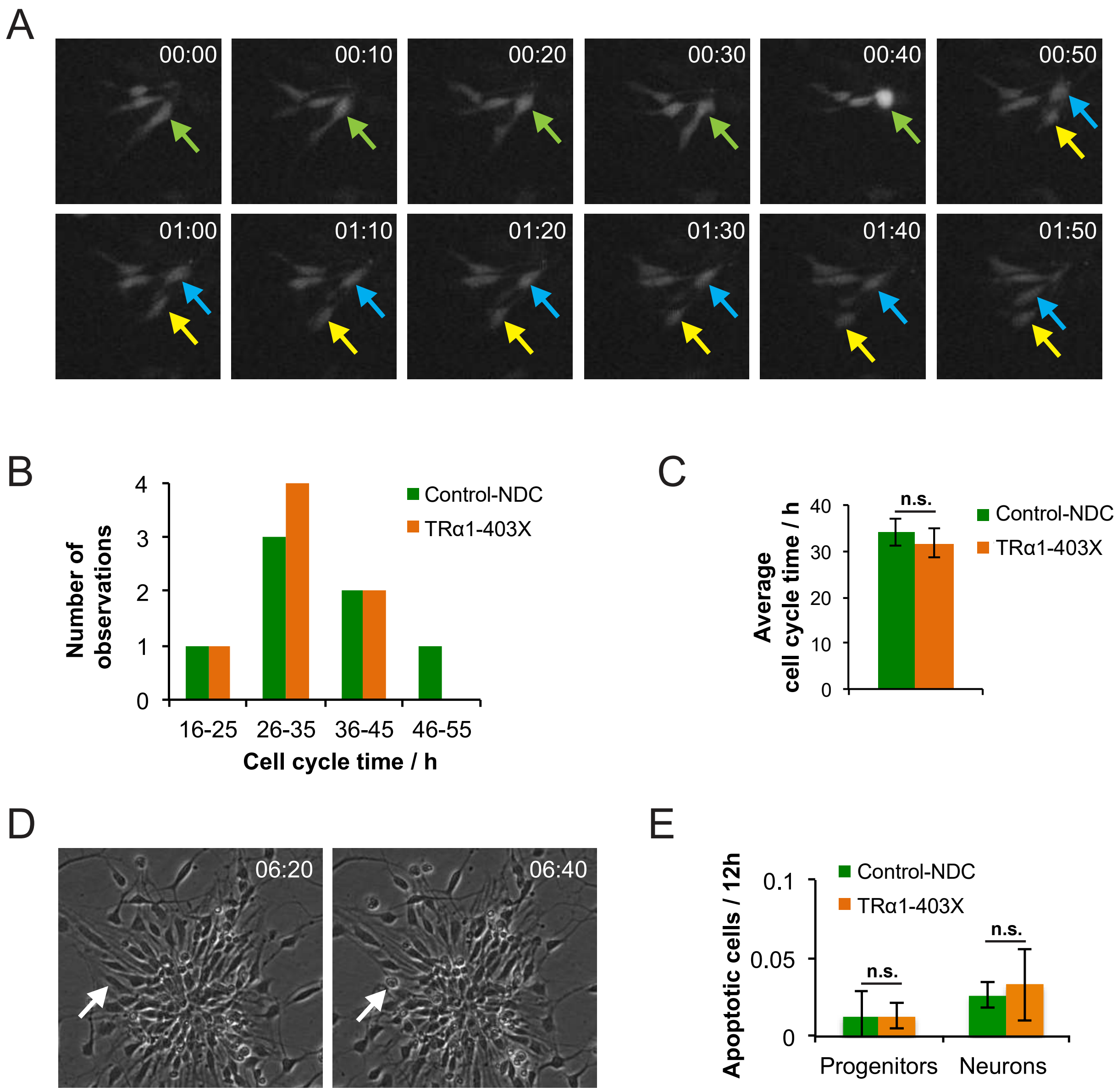
A
00:00
00:10
00:20
00:30
00:40
00:50
01:00
01:10
01:20
01:30
01:40
01:50
B
Number of observations
Control-NDC
TRα1-403X
16-25
26-35
36-45
46-55
Cell cycle time / h
C
Average cell cycle time / h
n.s.
Control-NDC
TRα1-403X
D
06:20
06:40
E
Apoptotic cells / 12h
Control-NDC
TRα1-403X
n.s.
n.s.
Progenitors
Neurons

Figure S5: Clonal composition in control and *THRA* mutant cortical cells

(A) The size distribution of fully Ki67⁻ clones at 6 and 10 days post mixing (dpm), and model fit. Data points represent the average of three control and three *THRA* mutant inductions; error bars indicate SEM.

(B) The joint distribution of Ki67⁺ and Ki67⁻ cells in *THRA* mutant and control clones at 2, 6 and 10 days post mixing at age 30 or 40 days. Colours indicate the fraction of clones at each coordinate. Results represent the average of three control and three *THRA* mutant lines.

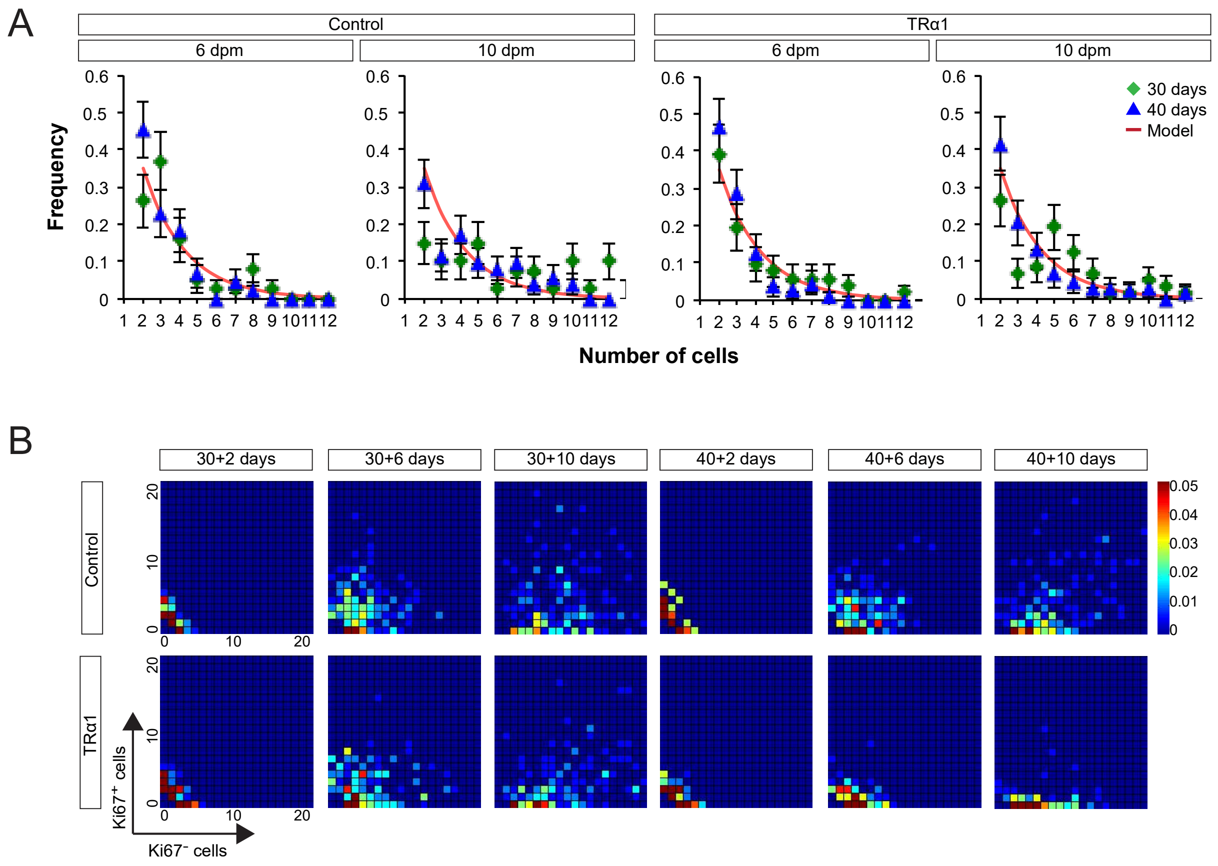

Figure S6: Increased expression of neuronal layer markers in *THRA* mutant cultures

Expression of TBR1 and CTIP2 was quantified in 3-5 images containing between 384 and 916 nuclei each from one induction per cell line (* $p<0.05$, two-sided Student's t-test comparing a total of 4 control and 12 *THRA* mutant images). Error bars indicate SEM.

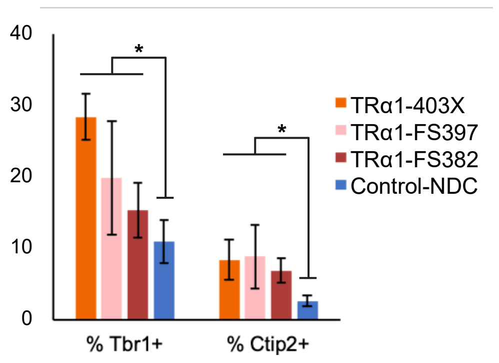

Figure S7: A minimal model for rosette formation *in vitro*

(A) Control cultures (NDC) dissociated at day 25 and treated with 10 μM 1-850 or DMSO (control) for five days. Treatment with 1-850 reduced the number of rosettes per field of view, which were identified as radially arranged nuclei around foci of atypical protein kinase C (yellow circles). The proportion of cycling cells was also reduced. Scale bars, 100 μm. * $p<0.05$, ** $p<0.01$ (Student's t-test).

(B) Dissociated *THRA* mutant (P1) and control (NDC) cultures were plated onto micropatterned chips and live imaged for a period of 48 hours, starting at 24 hours after plating. Shown are 10 representative traces of cellular movement, with no evidence for directionality. *THRA* mutant progenitors exhibited slightly higher average migration speeds than controls, which may be the result of beginning rosette assembly in control cultures constraining movement.

(C) Rosette formation was reproduced by a simple computational model in which cells – approximated as rods of length 20 μm – undergo random movement at a speed of 17 μm per frame (10 min) in two dimensions, and attach to each other at their apical ends when within 17 μm. Once formed, attachments are assumed to persist indefinitely. Over time, this dynamic leads to rosette formation *in silico* (top; purple dots represent the apical ends of cells, and black dots represent two or more apical ends that have attached to each other). The number of rosettes and the distribution of rosette centres observed *in vivo* are statistically reproduced by the model (bottom; NDC, n=50; simulation, n=100).

(D) In control cultures, N-cadherin, γ-tubulin and PKCζ and were localised to the apical end of progenitor cells in rosettes. In THRA mutants, expression of these factors was diffuse or greatly reduced. Scale bars, 50 μm.

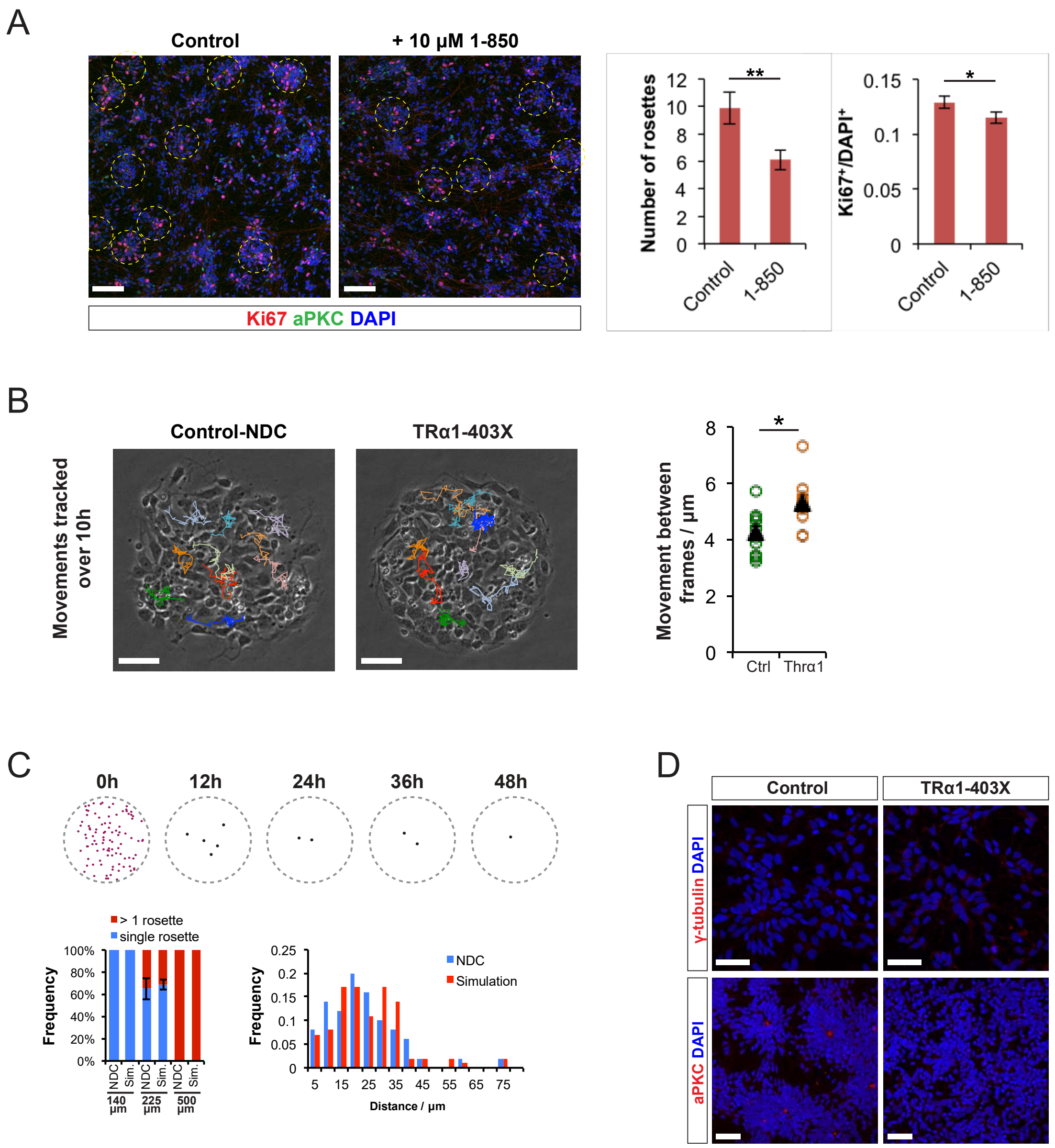

A
Control
+ 10 μM 1-850
Ki67 aPKC DAPI
Number of rosettes
**
Control
1-850
Ki67+/DAPI+
*
B
Control-NDC
TRα1-403X
Movements tracked over 10h
Movement between frames / μm
*
Ctrl
Thrα1
C
0h
12h
24h
36h
48h
> 1 rosette
single rosette
Frequency
NDC
Sim.
140 μm
225 μm
500 μm
NDC
Simulation
Distance / μm
D
Control
TRα1-403X
γ-tubulin DAPI
aPKC DAPI

Supplementary Tables

Table 1: Characteristics of RTHα patients who underwent neurocognitive and neuroimaging assessment

	**P1**	**P2**	**P3**	**P4**
THRA Mutation	E403X	F397fs406X	F397fs406X	A382PfsX7
Age at Diagnosis	6 years	12 years	47 years	48 years
Age at phenotyping	8.5 years	13.5 years	47 years	49.5 years
Gender	Female	Female	Male	Female

Table 2: Summary of neurological findings in patients

	P1	P2	P3	P4
Slow to initiate movement	++	+	+	+++
Abnormal Gait (broad based, ataxic)	+	+	+	+++
Abnormal Speech (slow, dysarthric, thick quality)	++	+	+	+++
Dysdiadochokinesis	+	+	+	+
Intention tremor	-	-	-	+
Slow Relaxing reflexes	+	+	-	-
Fine and Gross Motor incoordination	++	+	-	+++
Hypotonia	+	-	-	-

+++ severe, ++ moderate, + mild.

Table 3: Summary of developmental milestones in patients

	P1			P2	P4
	Age 16 months	Age 20 months	Age 27 months		
Gross Motor Skills	7-9 months	12-13 months	15-18 months	Sit 9 months, walk 19 months	Walking at 4 years
Fine Motor Skills	13-18 months	18-21 months	24 months		"poor"
Communication	11-15 months	11-18 months	11-18 months	First words 19-20 months, short sentences 2-4 years	First spoke at 3.5 years

No developmental data are available for P3.

Table 4: Summary of neuropsychological parameters in patients

Test Domain	P1	P2	P3	P4
*** Nonverbal IQ**	Low	Low	Low	Exceptionally Low
**+ Visual Perception**	Average	Low Average	Average	Exceptionally Low
**+ Visual Motor Integration**	Low	Low	Exceptionally Low	Exceptionally Low
**+ Motor Coordination**	Exceptionally Low	Exceptionally Low	Exceptionally Low	Exceptionally Low
**§ Finger Dexterity Dominant Hand**	Exceptionally Low	Exceptionally Low	Exceptionally Low	----
**§ Finger Dexterity Non-Dominant Hand**	Exceptionally Low	Exceptionally Low	Exceptionally Low	----

*** Wechsler scales (P1 and P2 WISC-IV, P3 WAIS-IV, P4 WASI-II)**
+ Beery-Buktenica Developmental Test of Visual Motor Integration Sixth Edition
§ Annett Peg Sorting Test

Table 5: N-Acetylaspartate/Total Creatine (NAA/Cr) ratio measured by MRS

	**P2**	**P3**	**P4**	**Range of Control Values**
Frontal White Matter	1.77	1.57	1.54	2.19-2.5
Thalamus	1.91	2.06	1.94	2.09-2.24

Supplementary Methods

Neuroimaging

Whole brain MRI scanning was performed on a 1.5T Siemens Magnetom Avanto Scanner (Siemens, Erlangen, Germany) at Great Ormond Street Hospital, London, UK. Axial T2 weighted fluid attenuated inversion recovery (FLAIR) images and coronal T1 weighted scans were compared with healthy age-matched controls. Diffusion Tensor Imaging was performed as described previously (52), and results compared with data from 20 age and gender-matched controls.

Proton magnetic resonance spectra (acquired using a point-resolved spectroscopy (PRESS) sequence with TR=1600ms, TE=135ms and a voxel size of 2 x 2 cm) of frontal white matter and thalamus of patients were analysed for N-acetylaspartate (NAA) and creatine plus phosphocreatine (Cr), with the results expressed as a ratio (NAA/Cr) and compared to a reference range from healthy, age-matched control subjects (53–57).

Pluripotent stem cell culture and directed cortical differentiation

Human control PSCs (H9 ESCs,WiCell Research Institute; NDC1.2 iPSCs (58); NAS6 iPSCs (T. Kunath, Edinburgh) and *THRA* mutant iPSCs were cultured under feeder-free conditions in Essential 8 Medium on Geltrex-coated tissue culture plates (Life Technologies). Neural induction was performed as previously described (21). Briefly, confluent PSCs were incubated for 12 days with a 1:1 mixture of N2 and B27 medium supplemented with 100 nM LDN-193189 (Stemgent) and 10μM SB431542 (Tocris). The neuroepithelial sheet was then broken up with Dispase (Life Technologies), and plated onto laminin-coated plates with N2B27 medium supplemented with 20 ng/ml FGF2 (Peprotech) for 4 days. From day 16 of induction, cells were maintained in N2B27 medium up to 60 days.

FACS sorting

Cells were dissociated with Accutase (Sigma-Aldrich, cat. no. A6964) for 10 minutes at 37°C with triturating every 5 minutes. Following incubation in DNaseI at 100 units/ml for 10 minutes, cells were strained through a 50μm cell strainer and counted. For each sample, 10^6 cells were collected by centrifugation and stained with fluorochrome-conjugated antibodies (BD Biosciences) in 100 μl neural maintenance medium containing 0.5% FBS (Hyclone, Fisher Scientific, cat. no. 12359792) and 0.5 mM EDTA (Life Technologies, cat. no. 15575-020) for 30 minutes on ice. Stained cells were sorted using an S3 Cell Sorter (Bio-Rad) in N2B27 medium with 10 μM ROCK inhibitor Y-27632 dihydrochloride (Tocris, cat. no. 1254).

RT-PCR

Total RNA from cortical cultures was isolated using Trizol (Sigma), and reverse-transcribed to cDNA using random hexamer primers (Applied Biosciences). Semi-quantitative RT-PCR was performed using primers against *FOXG1*, *PAX6* and *GAPDH*, and visualized in a Gel Doc XR+ Imager (Biorad).

Western blotting

Protein was extracted from overnight frozen cell pellets at -80°C, using Cell Extraction Buffer (Invitrogen) containing 1x complete mini protease inhibitor (Thermo Scientific). The insoluble fraction was removed by centrifugation at 12,000 g for 15 min at 4°C. Protein concentration was determined using Precision Red Advanced Protein Assay (Cytoskeleton, Inc.) according to manufacturer's instructions. 10-30 μg of total protein were mixed with NuPAGE LDS Sample Buffer and NuPAGE Reducing Agent to a final concentration of 1x. Samples were heated to 95°C for 10 min and cooled on ice prior to loading on pre-cast Bis-Tris gels and run at 200 V for at least 1 h. Proteins were transferred from the gel onto pre-soaked LiCOR PVDF membranes for 1h at 100V in 1x Tris-Glycine buffer with 20% methanol. Following transfer, the membrane was washed in TBS with 0.1% Tween (TBST), then blocked for 1 h at room temperature in TBST and 3% BSA (TBST+B). Membranes were incubated with β-actin and TRα1 (Pierce, cat. no. PA1-211A) primary antibodies diluted in TBST+B overnight at 4°C. The membrane was then washed five times with TBST and incubated in secondary antibody diluted 1:5,000 in TBST+B for 2 h at room temperature. Following a further five washes in TBST, the membrane was visualised on an Odyssey LiCOR machine. Band intensity was measured using Image Studio software (Li-COR), and normalised to β-actin.

Electrophysiology

For electrophysiological recordings, cortical neurons were incubated with artificial cerebral spinal fluid (aCSF) containing 125mM NaCl, 25mM $NaHCO_3$, 1.25mM NaH_2PO_4, 3mM KCl, 2mM $CaCl_2$, 25mM glucose and 3mM pyruvic acid, equilibrated in 95% O_2 and 5% CO_2. Borosilicate glass electrodes with resistance of 6-10MΩ were filled with an artificial intracellular solution, containing 135mM potassium gluconate, 7mM NaCl, 10mM HEPES, 2mM Na_2ATP, 0.3mM Na_2GTP and 2mM $MgCl_2$, and positioned over a cortical neuron to form a whole-cell patch. Recordings were made using a Multiclamp 700A amplifier (Molecular Devices), and signals were sampled and filtered at 20kHz and 6kHz respectively. A low-pass

Gaussian filter was applied to filter out high frequency noise. To detect sodium and potassium currents, step depolarisations were applied from a holding potential of -80 mV up to +40 mV in voltage clamp mode. To detect action potentials, stepwise current injections were applied from -10 pA up to +60 pA in steps of 5-10 pA in current clamp mode. To measure miniEPSCs, cells were held at -70 mV and current was recorded for 2-5 min.

Calcium imaging

Cells were incubated in N2B27 medium containing 3.2 μM calcium indicator Oregon Green 488 BAPTA and 0.01% v/v Cremaphor EL for 1 h at 37°C with 7% CO_2 in the dark. Cells were then incubated in N2B27 medium for a further 30 min at 37°C with 7% CO_2 in the dark. Neural maintenance medium was replaced with aCSF before imaging, and cells were placed in a microscope chamber heated to 37°C with 5% CO_2. Calcium activity in cultures was recorded at 10 Hz on a Deltavision fluorescence microscope with an EMCCD camera (Applied Precision) and using softWoRx 5.0.0 software. Three to five two-minute videos were recorded from one to two independent inductions from each cell line. For pharmacological experiments, cells were incubated in aCSF containing 50μM CNQX (Tocris Bioscience, cat. no. 0190) or 1μM tedrodotoxin (Tocris Bioscience, cat. no. 1078). Cells were imaged 10 min after drug application and 10 min after washout.

Movies were created from individual image files using ImageJ, and analysed with custom written software (based on templates written by Hugh Robinson, PDN, Cambridge) in Matlab (MathWorks). To quantify the level of calcium activity, the total calcium activity A_{Ca} was calculated as the change in fluorescent signal (ΔF/F) integrated over time, as

$$A_{Ca} = \int_t \left|\frac{d}{dt}\Delta F/F\right|.$$

Results from 3-5 videos per induction were averaged.

Immunofluorescence and imaging

For immunocytochemistry, cells were fixed with 4% paraformaldehyde in PBS and processed for immunofluorescence staining. Primary antibodies used: α-acetylated α tubulin (Sigma-Aldrich T6793), α-atypical PKC (Santa Cruz sc-216), α-βIII tubulin (Covance PRB-435P), α-cleaved Caspase 3 (Cell Signaling 9661L), α-Ctip2 (Abcam ab18465), α-γ-tubulin (Abcam ab11316), α-Ki67 (BD 550609), α-MAP2 (Abcam ab5392), α-Nanog (R&D Systems AF1997), α-N-cadherin (Abcam ab18203), α-Nestin (Abcam ab22035), α-Oct4 (Abcam ab19857), α-PAX6 (Covance PRB-278P), α-phospho-histone H3 (Abcam ab10543),

α-SATB2 (Abcam ab51502), α-SSEA4 (Santa Cruz sc-21704), α-TBR1 (Abcam ab31940), α-TRα1 (Pierce PA1-211A), α-Tra-1-60 (Abcam ab16288), α-Vimentin (Abcam ab8973), α-vGlut1 (Synaptic systems 135 303). Immunostained samples were imaged using a Leica SP5 inverted confocal microscope. Quantifications were performed using Volocity and ImageJ. For quantification of Pax6 expression within and outside of progenitor cell rosettes, images were binarized using Otsu's method. Rosette areas were selected manually based on morphology, and the binarized DAPI and Pax6 signals were integrated inside and outside of rosettes. Relative expression (Pax6/DAPI) was averaged across 8-9 images from 2-3 inductions per cell line.

Clonal lineage analysis

Third generation replication-incompetent lentivirus was produced by calcium phosphate transfection of HEK293T cells, using pBOP-GFP plasmids combined with packaging plasmids pRSV-Rev, pMDLg/pRRE and pMD2.G. For clonal lineage analysis, cortical progenitor cells were infected with the lentivirus at high titre before isolation of the $CD271^{-}CD44^{-}$ population by FACS. At day 30 and day 40, sorted progenitors were mixed with unlabeled controls cultures and plated at a density of $1.0x10^5$ cells/cm^2. Cultures were fixed and immunostained at 2, 6 and 10 days after plating.

Micropattern chip cultures

Neural progenitor cells were dissociated using Accutase, washed once in N2B27 and strained through a 50 µm cell strainer before counting. 10^6 cells were plated onto laminin-coated CYTOOchips in neural maintenance medium containing 20 ng/ml FGF2. After 24 hours, the medium was replaced with neural maintenance medium without FGF2, and cultures were kept for 1-7 days.

Live imaging

For live imaging, tissue culture dishes containing cells in N2B27 medium were placed in a BioStation CT (Nikon) at 37°C with 7% CO_2. Images were acquired in phase and green fluorescence channels at 10x or 20x magnification every 10 minutes for a period of 48-72 hours.

RNA sequencing

For RNA-seq library preparation, total RNA was extracted (as outlined above) from 3 control lines (H9, NDC1.2 and NAS6, 1 induction per line) and 3 *THRA* mutant lines (P1, P2 and P4, 3 independent inductions per line), all at day 12 of *in-vitro* cortical induction. A total of 2 µg

of RNA were purified and in-column DNase-treated to remove traces of contaminating chemicals and DNA, respectively, using RNeasy Mini kit (QIAGEN). Purified RNA samples were quantified using Qubit RNA Broad-range assay Kit (Thermo Fischer Scientific). Total RNA-seq libraries were prepared from 1 μg of purified RNA using the TruSeq Stranded Total RNA LT with Ribo-Zero (Human/Mouse/Rat, Set A) sample preparation kit (Illumina) according to the manufacturer's instructions. Libraries were pooled together and single-end sequenced with a read-length of 50 bp on an Illumina HiSeq1500 (Illumina). Approximately $3*10^7$ reads were generated per each multiplexed library.

For the expression profiling, read counts were generated for each of the RefSeq transcripts, using exon positions from UCSC hg19. RPKMs (reads per kilobase per million) were calculated by normalizing read counts for each transcript by the transcript length and the total number of reads in the corresponding sample, and summed for each gene symbol. The RPKMs for the samples were then clustered using unsupervised, hierarchical clustering with a Ward distance measure. Counts per million (CPM) and differentially expressed transcripts were called using edgeR (59). Transcripts remained in the analysis if they had CPM>1 in all CK or control samples. The log2 fold change (logFC) and the false discovery rate (FDR) were then generated using the filtered group.

Gene expression profiles were clustered using the GeneE software, based on Pearson's correlation. Gene ontology (GO) analysis was performed using the PANTHER database (www.panther.org). Enriched GO terms with at least five represented genes and $p<0.05$ were included in the analysis.

Computational model of human cerebral cortex neurogenesis

The modeling scheme used to analyse the clonal lineage data was based on the findings of a recent *in vivo* genetic labeling study of cortical neurogenesis in mouse, which showed that cortical radial glia progenitor cells (RGs) transit from a symmetrical proliferative phase to a neurogenic phase, in which they asymmetrically give rise to intermediate progenitor cells (IPCs) with variable but limited neurogenic potential (32). In primates, the progenitor cell compartment is more complex, including ventricular and outer radial glia that interconvert between different subtypes (60,61). While a comprehensive quantitative description of human cortical development was thus not feasible, this analysis aimed to identify robust differences in progenitor cell dynamics between Thrα1 mutant and control cell lines. We considered the evolution of clones in control lines first and, in a second step, compared these findings to the Thrα1 mutant clonal data.

Clonal behaviour in control cultures

As RGs are defined by their long-term self-renewal potential, clones that have lost all Ki67$^+$ cells by 10 days post-mixing (dpm) are assumed to derive from IPCs. The distribution of Ki67$^-$ clones was consistent with a model in which IPCs cycle at a constant rate λ_I and, on each division, self-renew asymmetrically with probability q, or differentiate symmetrically with probability 1-q. The distribution of cell cycle times was approximated as a Gamma distribution with scale parameter θ_I = 0.5; within a reasonable range, the exact choice of scale parameter proved inconsequential. The size distribution of Ki67$^-$ clones labelled at day (D) 30 and D40 in control lines was then well approximated with q = 0.65 ± 0.05 and λ_I = 0.65 ± 0.05 per day, consistent with IPC behaviour remaining constant over the time period considered here (see SI Appendix, fig. S4A). As expected, by 10 dpm, this model predicts that all IPC-derived clones are fully differentiated. From the fraction of Ki67$^-$ clones at 10 dpm (see Fig. 6A), and assuming that RGs and IPCs are labelled with equal efficiency, it followed that around 25% of cycling cells at day 30, and 31% at day 40, are IPCs.

In mouse neocortical development, RGs transition through a series of symmetric proliferative divisions before entering a phase of asymmetric divisions into IPCs or neurons (32). To determine whether a similar sequence of events could be distinguished in the human data, we considered the joint distribution of Ki67$^-$ cells, which are mostly or exclusively neurons, and Ki67$^+$ cells, which include RGs and IPCs, in clones (see SI Appendix, fig. S4B). In the D30 data at 6 dpm, some clones contained up to seven Ki67$^+$ and no differentiated cells, indicating that at least a proportion of cycling cells are still proliferating symmetrically. By 10 dpm, however, no clones were observed that consisted of more than seven Ki67$^+$ cells but no neurons. Therefore, progenitors that initially divided symmetrically are producing neurons by 10 dpm. At the same time, a similar frequency of seven-cell Ki67$^+$-only clones was also observed at 10 dpm in the day 40 data. Given the asymmetric division pattern of IPCs, this suggests that RGs do not transition unidirectionally from symmetric to asymmetric divisions.

Instead, we probed whether their dynamics were consistent with a model in which the choice between symmetric and asymmetric divisions is made stochastically at the level of individual RGs. In this model, RGs cycle at a constant rate λ_R and, on each division, self-renew symmetrically with probability r_{PP}, asymmetrically produce an IPC with probability r_{PD}, or produce two IPCs with probability 1-r_{PP}-r_{PD}. Again, the cell cycle times were taken to follow a Gamma distribution. Since the scale parameter, θ_R, significantly affected the outcome in this case, it was included as a parameter to fit.

Fitting the model by weighted least squares to the observed average sizes of 'persisting' clones, meaning clones that retain at least one Ki67$^+$ cell, good accordance was achieved for the D30 control data with $\lambda_R = 0.47 \pm 0.02$ per day, r_{PP}= 0.30 ± 0.05, r_{PD}= 0.65 ± 0.05, and θ_R = 1.1 ± 0.3 (Fig. 6B). Importantly, with these parameters, the model predicts the size distribution of 'persisting' clones at 6 dpm and 10 dpm. The total clone size distribution, including fully differentiated clones, was also well predicted (Fig. 6C).

The same parameter choice resulted in a good approximation of the clonal data from control cultures infected with GFP-lentivirus at day 40 (Fig. 6B,C), suggesting that any change in progenitor cell behaviour over this time period is small.

Clonal behaviour in TRα1 mutant cultures

The distribution of Ki67$^-$ clones in TRα1 cultures was indistinguishable from control cultures, suggesting that IPC behaviour is not affected by TRα1 mutations (see SI Appendix, fig. S4A). However, the fraction of fully differentiated clones in the D30 data was higher than in controls; only 66% of clones retained Ki67$^+$ cells at 10 dpm (see Fig. 6A). Assuming, as before, that these persisting clones are derived from RGs, the fitting procedure was repeated for the TRα1 mutant data at D30, using the cell cycle parameters λ_R and θ_R found from the control lines. A good approximation of the average 'persisting' clone sizes was obtained with r_{PP}= 0.15 ± 0.03 and r_{PD}= 0.80 ± 0.05 (Fig. 6B). With these parameters, the model correctly predicts the size distribution of 'persisting' clones and consequently the total clone size distribution (Fig. 6C).

In the D40 clonal data, only 20% of clones contained cycling cells at 10 dpm, and the average clone sizes were markedly decreased (Fig. 6A,B). With r_{PP}= 0.05 ± 0.05 and r_{PD}= 0.10 ± 0.05, the average clone sizes were still well approximated (Fig. 6B); a satisfactory approximation of the clone size distribution was also obtained (Fig. 6C). The fate choice probabilities r_{PP} and r_{PD} are therefore significantly reduced in TRα1 mutant compared to control cultures at D40.

Premature neurogenesis and progenitor depletion

To summarise, the control clonal data at D30 and D40 are well approximated by a highly simplified model of cortical development. In this model, RGs cycle on average once every 51 ± 2 hours. On each division, they choose stochastically between symmetric self-renewal with a probability of 30 ± 5 %, asymmetric division with a probability of 65 ± 5 %, or symmetric differentiation into IPCs. IPCs themselves cycle on average once every 37 ± 3 hours. 65 ± 5 % of IPC divisions are asymmetric and the remainder are symmetric differentiating divisions into two neurons, which results in virtually all IPC-derived clones differentiating fully by 10 dpm.

The TRα1 mutant clonal data is well described by the same model with the same cell cycle parameters, suggesting that the unidirectional lineage hierarchy (RGs producing IPCs which in

turn give rise to Ns) and characteristic cellular properties are not affected by the mutations. Instead, the observed clone sizes and compositions are consistent with a change in RG fate choices upon division. At D30, the probability of symmetric self-renewal of RGs is only 15 ± 3 %, while 80 ± 5 % of divisions are asymmetric. At D40, the vast majority of divisions are symmetric differentiating divisions into two IPCs. Consistently, the estimated proportion of RGs in cultures decreases much faster in TRα1 mutant lines, reaching 20% at day 40.

The cell cycle times estimated from the clonal data agree well with earlier *in vitro* estimates based on clone sizes at 2 dpm and BrdU incorporation, as well as previously reported results from non-human primates (62). As a further consistency check, dissecting out the $Ki67^+$ cell content of clones, the model provides an independent prediction of the progenitor cell number within clones (see Fig. 6B).

While a more complex model might provide an equally good, or better, description of the data, these results suggest that the simplistic model introduced here contains the minimal necessary rules governing stem cell dynamics in TRα1 mutant and control cortical cultures. Importantly, the dramatic difference in clonal dynamics is largely accounted for by the premature differentiation of RGs into IPCs, without any changes to cell cycle kinetics or lineage hierarchy. Over time, the decreased self-renewal of RGs leads to a markedly different output predicted per initially labelled RG over the 20-day period from day 30 to day 50. In control cultures, on average, the model predicts that one RG labelled at day 30 gives rise to approximately 8.2 RGs, 8.1 IPCs, and 52.2 neurons by day 50. In stark contrast, in TRα1mutant cultures, the average RG labelled at day 30 results in less than 0.1 RGs, 1.2 IPCs, and 28.1 neurons over the same time period. Not only are neurons generated prematurely by TRα1 mutant progenitor cells, but the pool of RGs with long-term self-renewal potential is also depleted much earlier than in controls, leading to a reduction in the overall number of neurons produced during cortical development.

References

52. Gibbard CR, et al. (2013) White matter microstructure correlates with autism trait severity in a combined clinical-control sample of high-functioning adults. *NeuroImage Clin* 3:106–114.

53. Filippi CG, Uluğ AM, Deck MDF, Zimmerman RD, Heier LA (2002) Developmental delay in children: Assessment with proton MR spectroscopy. *Am J Neuroradiol* 23(5):882–888.

54. Komoroski RA, et al. (2004) Brain metabolite concentration ratios in vivo: Multisite reproducibility by single-voxel1H MR spectroscopy. *Magn Reson Imaging* 22(5):721–725.

55. Ozturk A, et al. (2009) Proton MR spectroscopy correlates of frontal lobe function in healthy children. *Am J Neuroradiol* 30(7):1308–1314.

56. Safriel Y, Pol-Rodriguez MA, Novotny EJ, Rothman DL, Fulbright RK (2005) Reference values for long echo time MR spectroscopy in healthy adults. *Am J Neuroradiol* 26(6):1439–1445.

57. Staffen W (2005) Magnetic Resonance Spectroscopy of Memory and Frontal Brain Region in Early Multiple Sclerosis. *J Neuropsychiatr* 17(3):357–363.

58. Israel MA, et al. (2012) Probing sporadic and familial Alzheimer's disease using induced pluripotent stem cells. *Nature* 482(7384):216–220.

59. Robinson MD, McCarthy DJ, Smyth GK (2009) edgeR: A Bioconductor package for differential expression analysis of digital gene expression data. *Bioinformatics* 26(1):139–140.

60. Hansen D V, Lui JH, Parker PRL, Kriegstein AR (2010) Neurogenic radial glia in the outer subventricular zone of human neocortex. *Nature* 464(7288):554–561.

61. Betizeau M, et al. (2013) Precursor diversity and complexity of lineage relationships in the outer subventricular zone of the primate. *Neuron* 80(2):442–57.

62. Otani T, Marchetto MC, Gage FH, Simons BD, Livesey FJ (2016) 2D and 3D Stem Cell Models of Primate Cortical Development Identify Species-Specific Differences in Progenitor Behavior Contributing to Brain Size. *Cell Stem Cell* 18(4):467–480.
